# Herbal Remedies as Potential in Cartilage Tissue Engineering: An Overview of New Therapeutic Approaches and Strategies

**DOI:** 10.3390/molecules25133075

**Published:** 2020-07-06

**Authors:** Constanze Buhrmann, Ali Honarvar, Mohsen Setayeshmehr, Saeed Karbasi, Mehdi Shakibaei, Ali Valiani

**Affiliations:** 1 Musculoskeletal Research Group and Tumour Biology, Chair of Vegetative Anatomy, Institute of Anatomy, Faculty of Medicine, Ludwig-Maximilian-University Munich, Pettenkoferstrasse 11, D-80336 Munich, Germany; constanze.buhrmann@med.uni-muenchen.de; 2 Department of Anatomical Sciences, School of Medicine, Isfahan University of Medical Sciences, Isfahan 73461-81746, Iran; honarvarali666@yahoo.com (A.H.); m_setayeshmehr@med.mui.ac.ir (M.S.); 3 Biomaterials Nanotechnology and Tissue Engineering Group, Department of Advanced Medical Technology, Isfahan University of Medical Sciences, Isfahan 73461-81746, Iran; karbasi@med.mui.ac.ir

**Keywords:** herbal remedies, cartilage, tissue engineering, osteoarthritis, curcumin, icariin, pomegranate, ginger, avocado/soybean unsaponifiables, resveratrol

## Abstract

It is estimated that by 2023, approximately 20% of the population of Western Europe and North America will suffer from a degenerative joint disease commonly known as osteoarthritis (OA). During the development of OA, pro-inflammatory cytokines are one of the major causes that drive the production of inflammatory mediators and thus of matrix-degrading enzymes. OA is a challenging disease for doctors due to the limitation of the joint cartilage’s capacity to repair itself. Though new treatment approaches, in particular with mesenchymal stem cells (MSCs) that integrate the tissue engineering (TE) of cartilage tissue, are promising, they are not only expensive but more often do not lead to the regeneration of joint cartilage. Therefore, there is an increasing need for novel, safe, and more effective alternatives to promote cartilage joint regeneration and TE. Indeed, naturally occurring phytochemical compounds (herbal remedies) have a great anti-inflammatory, anti-oxidant, and anabolic potential, and they have received much attention for the development of new therapeutic strategies for the treatment of inflammatory diseases, including the prevention of age-related OA and cartilage TE. This paper summarizes recent research on herbal remedies and their chondroinductive and chondroprotective effects on cartilage and progenitor cells, and it also emphasizes the possibilities that exist in this research area, especially with regard to the nutritional support of cartilage regeneration and TE, which may not benefit from non-steroidal anti-inflammatory drugs (NSAIDs).

## 1. Introduction

Currently, about 200 joint diseases are characterized by the term “osteoarthritis” (OA), also known as degenerative joint disease (DJD). OA is reported as the main cause of pain and disability in the joints of older people [1], and the joints most commonly affected by OA are the hip, knee, hand, and spine [2,3]. In addition, it has been reported that 20 percent of adults in Western Europe and North America will be exposed to OA by 2030 [4], and OA can be expected to be a significant economic burden on health systems and medical facilities worldwide [5]. OA is predominantly defined and characterized by the microtrauma and degradation of articular cartilage [6], massive intra-articular inflammation with synovitis, arthritis, and changes in periarticular structures and subchondral bone tissue [7]. Though OA affects all structures of the synovial joint [8], the degeneration and degradation of articular cartilage is the main cause of clinical symptoms, making cartilage regeneration the focus of attention and the basis for long-term treatment success [9,10,11]. Up to now, the treatment of OA has concentrated on anti-inflammatory therapies (mainly non-steroidal anti-inflammatory drugs (NSAIDs) to relieve pain) and preventive measures to improve the lifestyle of the patient (weight control, exercise, and nutritional advice) [12,13]. However, common clinical treatments with chemical agents and synthetic drugs do not have a cartilage-regenerating effect and are associated with several undesirable side effects. Therefore, there is a significant need for alternative, better regenerative approaches for the success in the long-term treatment of OA [14].

The cartilage tissue belongs to the family of bradytrophic connective tissues. Due to its unique macro- and microstructural composition and its highly organized structure, cartilage tissue poses a great challenge for researchers who want to repair and regenerate this highly specific tissue [15,16]. The unique architecture of articular cartilage consists of chondrocytes that produce and are embedded in a cartilage-specific, highly organized extracellular matrix (ECM). The main component of this ECM is water (60–80%), which is closely bound to the macromolecular components that consist of 40–50% collagens (90% collagen type II: COL2A1) and 20–25% of various specialized proteoglycans (aggrecan, decorin, biglycan, and fibromodulin) [17]. The cartilage-specific ECM is not only synthesized by the chondrocytes, as they are also in close functional interaction with each other and their production in the chondrocytes is stimulated and directly influenced by highly sensitive microenvironmental conditions that stimulate cartilage homeostasis and repair [18]. 

The main problem is that although cartilage tissue has a basic regenerative potential, it actually contains additional progenitor cells, mesenchymal stem cells (MSCs), which are the basic prerequisite for adequate tissue regenration, since under pathological conditions only repair tissue develops [19,20,21]. However, the repair tissue is not capable of withstanding physiological stress on the cartilage tissue in the joints, resulting in joint degeneration, the impairment of joint use, dysfunction, deformation, pain, and a considerable impairment of the patient’s everyday life [15,16].

In fact, for appropriate chondrogenesis, MSCs require an adequate stimulus through the external application of growth factors and cytokines, such as transforming growth factor-β (TGF-β), insulin-like growth factor−1 (IGF−1), and bone morphogenic protein−6 (BMP−6) [22,23,24,25,26]. 

The term “tissue engineering” (TE) is defined as the ability to reconstruct mammalian tissue both in structure and function [27]. In fact, the broad and extensive field of TE has emerged as a promising new branch of modern medicine aimed at realizing mankind’s dream of developing the ability to reconstruct functional transplantable tissues [28,29]. In recent decades, modern medicine has greatly enhanced our capabilities and reached many milestones in improving TE approaches, including cartilage TE [30,31]. However, common treatment approaches such as endoprosthetics, multiple drilling, microfractures, autologous chondrocyte implantation (ACI), and joint replacement are associated with many side effects [32,33]. In addition, there are still many limitations in creating an adequate cellular microenvironment: The scaffolds used must be biocompatible, biodegradable, non-toxic, and have the desired mechanical properties, and the materials used can be expensive and have not yet been fully tested [25]. 

In the attempt to refine and improve TE approaches for cartilage tissue and to find new, better, safer, and cheaper alternatives, attention has been drawn to natural, non-toxic, and anabolic active components from nature [34,35,36,37]. In traditional medicine, such as Ayurveda medicine (the science of long life) or traditional Chinese and Indian medicine, herbal remedies have been used for centuries to treat many diseases, including joint diseases. More recently, the use of herbal remedies and their derivatives to treat joint problems, including OA, has also become widely accepted in modern Western medicine due to their proven anabolic, anti-inflammatory, health, and medical benefits [38,39,40,41,42]. Indeed, there are a wealth of studies on the efficacy of herbal ingredients and their use in the treatment of a number of diseases, including anti-inflammatory effects on chondrocytes [14,35,43,44,45]. A major advantage of using herbal remedies for prevention and therapeutic treatment is also that they are accessible to a broad range of people at relatively low cost, are safe to use, and have no undesirable side effects. Though a proper scientific assessment of herbal medicinal products is a challenge, many practices in modern delivery systems have investigated the use of herbal medicines in biotechnology to overcome rheumatoid arthritis (RA) and OA. Here, herbal medicinal products are studied as raw herbs, herbal mixtures, herbal fusions, finished herbal products, or over-the-counter drugs [46]. Various processes such as steaming, roasting, or cooking could improve the properties and their effects. The preparation and the method of application and administration of herbs is crucial for the final results. The basis for herbal preparations is powdered herbal materials or extracts, tinctures, and fatty oils from herbal materials produced by extraction, fractionation, purification, concentration, or other physical or biological processes. Indeed, the study of the appropriate use of herbal medicine and herbal chemicals in TE should be considered as a suitable and substantial approach to the repair and regeneration of articular cartilage [46]. Therefore, this literature review focuses on the potential of herbal remedies as anti-oxidant and anti-inflammatory agents and their potential for cartilage regeneration and TE therapy, as well as the prevention and treatment of joint diseases, with a focus on OA.

### Data Acquisition

**Figure 1:**
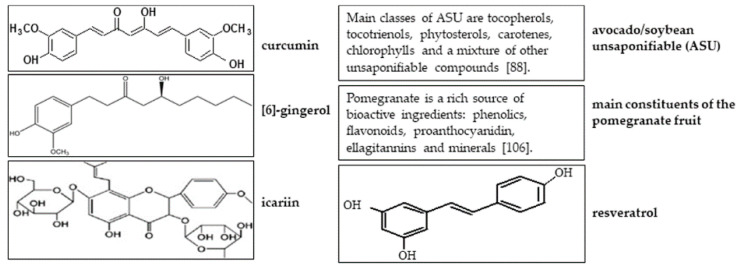
Structures of natural products with potential for cartilage regeneration, tissue engineering (TE), and the prevention and treatment of joint diseases.

The search was limited to scientific publications, journals, and textbooks. Both in vivo and in vitro studies were equally evaluated. The included data, abstracts, titles, and full texts were reviewed by two independent researchers to determine the relevance for inclusion in the study. The search terms used were ‘cartilage,’ ‘degenerative joint diseases,’ ‘tissue engineering,’ ‘herbal medicinal products,’ ‘nutraceuticals,’ ‘herbal remedies,’ and ‘osteoarthritis.’ Herbal remedy-related search terms were ‘icariin,’ ‘pomegranate,’ ‘ginger,’ ‘avocado/soybean unsaponifiable (ASU),’ ‘curcumin,’ and ‘resveratrol,’ as well as the term ‘herb’ and all its derivatives, combining the words with individual plant and herb names (Figure 1). No methodological search filter was applied. Scientists working in similar fields of study were contacted to evaluate the results and conclusions of the related literature. In case additional information was needed, the authors were contacted whenever possible.

Table 1, Table 2, Table 3, Table 4, Table 5 and Table 6 give a short overview of the reviewed studies. The accuracy of the data was validated by all authors. 

## 2. Curcumin

Natural polyphenol curcumin (diferuloylmethane) is the main active ingredient in turmeric (*Curcuma Longa,* also called curry) and derived from its rhizomes. Due to its anti-inflammatory and anti-oxidant properties, curcumin has a wide-range of medicinal applications including targeting cancers, diabetes, obesity, cardiovascular, pulmonary, neurological, and autoimmune diseases [47]. In fact, curcumin is used in traditional Indian Ayurvedic medicine to treat diarrhea, abdominal pain, nausea, intestinal gases, loss of appetite, jaundice, liver problems, gall bladder issues, and OA [43].

In OA treatment, curcumin has been recognized to target the catabolic-induced inflammatory effects of interleukin−1 beta (IL-1β) signaling, such as the matrix metalloproteinase−13 (MMP−13) up-regulation and inhibition of matrix synthesis, by exhibiting a chondroprotective effect [36]. Additionally, curcumin inhibited IL-1β- or tumor necrosis factor (TNF)-α- or TNF-β-induced inhibition of COL2A1, cartilage-specific proteoglycans (CSPGs), and β1-integrin synthesis in chondrocytes and blocked IL-1β-, TNF-α-, or TNF-β-induced apoptosis by caspase−3 inhibition [37,48,49]. Furthermore, curcumin was shown to suppress pro-inflammatory signaling pathways in human and rat articular chondrocytes by targeting IL-1β-induced transcription factor nuclear factor kappa B (NF-κB) activation and the suppression of NF-κB-regulated gene end-products such as cyclooxygenase−2 (COX−2) and MMP−9 [37,50]. Moreover, in IL-1β-stimulated cartilage explants, curcumin was able to block the IL−1β-induced loss of glycosaminoglycans (GAGs) [51]. Contrary to these findings, a comparative study investigating the effect of glucosamine (GlcN), curcumin, and diacerein in human chondrocytes found that curcumin was not as effective as GlcN and diacerein in stimulating expression of cartilage-specific genes such as aggrecan (AGC) and COL2A1, and it was even toxic at high concentrations [52]. Interestingly, a study by Buhrmann and co-workers (2010) showed a significant protective and stimulating effect of curcumin on the chondrogenesis of MSCs [53]. Here, a four-hour pre-treatment with curcumin significantly increased the production of COL2A1, CSPGs, and β1-integrin, as well as the activation of the mitogen-activated protein (MAP) kinase signaling pathway and the suppression of caspase−3 and COX−2. Moreover, in IL-1β-stimulated three dimensional co-cultures of MSCs and primary chondrocytes, curcumin inhibited the inflammatory effect of IL-1β and thereby stimulated adequate chondrogenesis [53]. These results underline the enormous potential of curcumin to suppress inflammatory signaling pathways and thereby create an appropriate anabolic and stimulatory microenvironment for chondrogenesis. 

Several studies have shown and evaluated curcumin-loaded electrospinning PLA (poly(lactic acid)) composite membranes and could demonstrate that curcumin dose-dependently decreased the average diameters of composite nanofibers, resulting in the superior anti-coagulation property of composite membranes compared to pure PLA membranes [54,55]. These strategies can support the specific potential regeneration of joint cartilage tissue [53,54]. Furthermore, Henrotin et al. (2010) summarized the protective effects of curcumin on articular chondrocytes, and they reported that curcumin protects chondrocytes from catabolic effects of IL-1β including MMP−3 up-regulation, the inhibition of COL2A1, and the down-regulation of β1-integrin expression. Indeed, curcumin blocks IL-1β-induced PG degradation, activator protein-1 (AP-1)/NF-κB signaling pathways, chondrocyte apoptosis, and the activation of pro-apoptotic proteins, such as caspase−3 [43]. Interestingly, a study by Golchin and co-workers (2019) showed that nanofibrous scaffolds fabricated by chitosan, poly(vinyl alcohol) (PVA), carbopol, and polycaprolactone using a dual electrospinning technique that had curcumin incorporated inside the chitosan (CS)/PVA fibers exerted better bactericidal activity against Gram-positive bacterial strains compared to Gram-negative strains and demonstrated significant compatibility with blood and fibroblast cells, making this curcumin scaffold combination a promising candidate for TE [56]. In addition, in a scaffold of curcumin and silk fibroin the GAG content and the analysis of mRNA expression indicated that the chondrocytes remained viable and continued to proliferate. Furthermore, the composition of the curcumin-silk scaffold showed a high biocompatibility and a favorable microenvironment for cartilage repair after transplantation in vivo [57]. In fact, several studies have shown that curcumin modulates the effects of pro-inflammatory cytokines such as the IL-1β-, or TNF-α, or TNF-β-induced suppression of specific cartilage ECM and signal molecules, such as integrins in human joint chondrocytes by antagonizing the IL-1β-, TNF-β-, or TNF-α-dependent up-regulation of pro-inflammatory biomarkers, such as MMP−9, caspase−3, and COX−2. More interestingly, curcumin blocks cytokines that promote the phosphorylation and nuclear translocation of the pro-inflammatory transcription factor subunit p65-NF-κB by the phosphorylation, degradation, and ubiquitination of IκB-α [37,48,49,57,58]. Taken together, these data suggest that curcumin has cartilage regeneration capacity as a naturally occurring anti-inflammatory multi-targeted compound for the treatment of OA or RA through the suppression of the master pro-inflammatory transcription factor NF-κB signaling pathways in chondrocytes.

## 3. Ginger

Ginger (*Zingiber officinale*) has been an important ingredient in Asian herbal medicine for centuries and is used especially for pain relief in diseases of the musculoskeletal system [59]. Moreover, ginger consists of a complex combination of biologically active ingredients, of which the compounds gingerols, shogaols, and parasols appear to account for the majority of its anti-inflammatory properties [60]. In addition, gingerol suppresses inflammatory degradation enzymes such as the nitric oxide (NO) synthase or COX-2, which have been shown to be regulated and promoted by the master transcription factor NF-κB [60]. In fact, the composition of ginger extraction varies according to the type of ginger, the maturity of the rhizome, the climate in which the plants are grown and harvested, and the process of extract preparation [61,62]. A meta-analysis of randomized controlled trials comparing oral ginger treatment with placebo in OA patients showed that ginger extracts had a variable and usually modest effect with a reduction in pain and a reduction in the disability of OA symptoms, and the extracts were well tolerated by the majority of patients [61]. Furthermore, in vitro analyses have shown that ginger can act as an inhibitor of COX, in particular of the inducible form of COX-2 rather than the constitutive form COX-1 [63]. In addition, ginger suppresses arachidonate 5-lipoxygenase, an enzyme of leukotriene biosynthesis, resulting in the suppression of the synthesis of inflammatory leukotrienes [64]. Indeed, in vitro studies have revealed that ginger also exerts its anti-inflammatory protective effects on chondrocytes and human synoviocytes by specifically decreasing the production of inflammatory mediators and chemokines induced by lipopolysaccharides (LPS), IL-1β, and/or TNF-α [65,66]. Furthermore, it has been shown that ginger root extract (GRE) significantly suppresses the production of NO and prostaglandin E2 (PGE_2_) in cartilage tissue grafts in vitro, and it linearly reduces production of PGE_2_ and NO in both normal and OA chondrocytes [66,67]. In addition, ginger extract efficiently suppressed the expression of pro-inflammatory cytokines, such as TNF-α, interleukin-6, and interleukin-8 mRNA levels, and it significantly reduced levels of both p38-mitogen-activated protein kinase (MAPK) and c-Jun *n*-terminal kinase phosphorylation, thereby reducing cartilage inflammations and degradation [68]. 

In a study by Hosseinzadeh and co-workers (2017), the toxicity of ginger on chondrocytes was investigated, and no cytotoxicity was observed even at high concentrations of ginger extract in chondrocyte cells [69]. In addition, they reported that ginger extract could significantly reduce IL-1β-induced oxidative stress, mitochondrial changes, and apoptosis [69]. Taken together, these data support the argument that ginger has positive prevention and pharmaceutical effects against OA and substantiates the enormous potential and promising role of ginger in stimulating an adequate chondrogenic microenvironment by suppressing inflammation due to arthritis, thus stimulating cartilage regeneration and TE.

## 4. Icariin

Icariin, commonly known as horny goat weed or Yin Yang Huo, is a bioactive flavonoid and phytoestrogen compound extracted from *Epimedium* that has been widely used in Asian countries practicing Chinese Traditional Medicine to treat conditions from hay fever and fatigue to atherosclerosis and osteoporosis [70]. 

Several reports have shown that icariin has great anti-inflammatory potential, and it has been investigated in a variety of diseases, including cancer and OA, by modulating autophagy and apoptosis; it ultimately been indicated to be a promising compound for cartilage TE in the treatment of OA [71,72]. In fact, in vitro studies with icariin have shown that it plays a protective role in OA by promoting chondrocyte differentiation, reducing chondrocyte apoptosis, and enhancing the secretion of specific ECM components by chondrocytes [73,74]. In addition, studies on chondrocytes of newborn mice showed that icariin pretreatment had protective effects against LPS-induced MMP, COX-2, and inducible nitric oxide synthase (iNOS) expression and reduced ECM production [75]. Interestingly, a study has demonstrated that higher concentrations of icariin (1 × 10^−5^ M) may have a better chondroprotective effect, leading to increased ECM production [74]. Here, the promotion of icariin in the synthesis of GAGs and collagen in chondrocytes could be due to its ability to upregulate the expression of the AGC, COL2A1, and SOX9 genes and to additionally downregulate the expression of the collagen type I gene in chondrocytes. Furthermore, icariin has been shown to be a safe and effective chondrocyte anabolic agent that stimulates chondrocyte proliferation and attenuates the breakdown of the ECM [75,76] by the activation of the miR-206 targeting of cathepsin K in rats, making it a promising candidate for supporting chondrogenesis in TE [77]. Moreover, in a study with MSCs from bone marrow, icariin effectively supported chondrogenesis by upregulating the mRNA expression levels and protein synthesis of COL2A1, AGC, and SOX9, as well as suppressing hypertrophic cartilage markers [78]. Several studies have investigated the potential of icariin in combination with biocompatible scaffolds as a basis for cartilage TE. Kankala and co-workers (2018) demonstrated the excellent adhesion rate and growth behavior of chondrocytes in in vitro cartilage cell proliferation experiments with a highly porous 3D scaffold based on cell-responsive polymeric inks, i.e., sodium alginate and gelatin (SA-Gel, 1:3 ratio), by a novel 3D printing method, and they found the porous architectures facilitated the efficient distribution of chondrocytes with only a few remaining on the surface. Interestingly, they found that icariin addition at a concentration of 10 μg/mL significantly enhanced the proliferation and viability of chondrocytes, indicating that icariin may have potential in engineering complex cartilage tissue constructs toward applications in cartilage TE [79]. In another study, icariin in combination with a hyaluronic acid–icariin hydrogel compound showed controlled drug release and good cytocompatibility, suggesting this combination as a potential scaffold for cartilage TE [80]. In addition, a study by Liu and co-workers in 2018 investigated the release behavior of icariin from gelatin/hyaluronic acid (gel/HA) microspheres and found that icariin had chondroprotective potential in a rat model of dexamethasone-induced cartilage lesion by the stimulation of miR-206, which acts on cathepsin K [77]. Interestingly, it has been further shown that chondrocytes encapsulated in icariin–HA/Col hydrogel showed a tendency to aggregate into larger clusters. The expression level of chondrogenic genes was remarkably upregulated, and the matrix synthesis of sGAG and type II collagen was significantly increased. Furthermore, the in vivo study showed that icariin–HA/Col constructs facilitated the reconstruction of the osteochondral interface in subchondral defects in rabbits. In the icariin–HA/Col group, the neocartilage layer contained more type II collagen, and the newly formed subchondral bone deposited abundant type I collagen. Taken together, these data suggest that the icariin–HA/Col hydrogel may be a promising scaffold for the reconstruction of an osteochondral interface, thereby promoting the restoration of the osteochondral defect and for TE [77,80,81]. Furthermore, in a chondrocyte alginate hydrogel 3D culture, icariin was able to increase proliferation, enhance chondrogenic marker expression, promote ECM synthesis, and markedly suppress catabolic gene expression including MMP-2, MMP-9, MMP-13, Adamts4, and Adamts5, possibly due to its role in activating HIF-1α [82]. Moreover, after transplantation into a mouse model, the icariin-loaded 3D hydrogel culture of chondrocyte alginate hydrogel significantly improved osteochondral defects and increased articular chondrocyte repair, as shown by higher histological scores [82]; additionally, in an in vitro study with a highly porous 3D scaffold based on sodium alginate and gelatin in 3D printing, icariin significantly promoted the proliferation of chondrocytes [79]. Additionally, in an in vivo study on the anterior cruciate ligament of the mouse in which a model of OA and a micro mass culture of mouse chondrocytes were induced, treatment with icariin resulted in an increased cartilage thickness; an upregulated expression of COL2A1; a reduced chondrocyte hypertrophy; a downregulated expression of collagen type X and MMP13; an upregulated expression of AGC, SOX9, and parathyroid hormone related proteins (PHrP); and a down-regulation of Indian hedgehog (Ihh) and genes regulated by Ihh [83,84,85]. 

Taken together, the results of these different in vitro and in vivo studies clearly indicate that the integration of icariin into different scaffolds and direct administration into the joint could be a very promising approach to improve cartilage regeneration and TE.

## 5. Avocado/Soybean Unsaponifiables

Avocado and soybean extract, known as avocado/soybean unsaponifiables (ASUs), is a natural remedy derived from avocado and soybeans [86]. It is a plant-based extract that consists of one third avocado oil and two thirds soybean oil [87]. Though its mechanism of action is not yet fully understood, it exerts an anti-inflammatory potential that prevents the degeneration of cartilage and joints and supports the regeneration of connective tissue [46]. Overall, it has been shown that ASU components exert anti-inflammatory and pro-anabolic effects in chondrocytes and enhance chondroprotective properties by stimulating the production of type II collagen and CSPGs [22,23]. It has been reported that ASUs’ most widely used commercial product for the treatment of OA is Piascledin, which contains extracts of avocado and soybeans in a 1:2 ratio [88,89]. Several in vitro studies have shown that Piascledin has an inhibitory effect on type II collagenase and prostaglandins, stimulates the synthesis of PGs and COL2A1, and reduces the synthesis of fibronectin, thereby improving cartilage formation [24,90]. Moreover, it has been shown that IGFBP-3-fibronectin interactions affect the IGF-I pathway, and it has been indicated that IGF-I is deposited in the chondrocyte matrix by binding to a complex of IGFBP-3 and intact fibronectin. This setup could play an important role in the control mechanisms of cartilage damage [24]. Additionally, Piascledin inhibits the effect of pro-inflammatory cytokines such as IL-1β and has a stimulatory effect on the synthesis of COL2A1 in chondrocytes and on TGF-β1 [91]. 

Furthermore, it has been shown that ASU can stimulate the expression of TGF-β1 and TGF-β2 in chondrocytes in vitro, and in vivo studies have shown that ASU increases levels of TGF-β1 and TGF-β2 in knee synovial fluid, thereby significantly reducing OA lesions. Interestingly, in an in vivo study, it was shown that ASU treatment showed significant symptomatic efficacy versus placebo in the treatment of OA, which occurred from the second month and even showed a sustained effect after the end of treatment [92,93,94]. Furthermore, ASU potently inhibits the production of IL-8 and PGE_2_, and it reverses inflammatory IL-1β effects on chondrocytes, underling the potential of ASU for attenuating the degenerative effects of IL-1β on cartilage [88]. 

The combination treatment of ASU with α-lipoic acid (LA) significantly suppressed LPS-, IL-1β-, or hydrogen peroxide (H_2_O_2_)-induced degenerative signaling pathways in equine chondrocytes in vitro, exerting significant anti-inflammatory and chondroprotective effects compared to controls [95]. Interestingly, the anti-inflammatory effects of ASU are not limited to chondrocytes, as they also target monocyte/macrophage-like cells in the synovial membrane [96].

A HPLC and gas chromatography (GC) mass spectrometry study showed that the ASU-mediated inhibition of IL-1β-induced MMP-3 activity, sulfate release, and PGE_2_ synthesis and that the ASU-mediated upregulation of GAG and collagen synthesis were dose-dependent [97]. A computer-assisted histomorphometry analysis of the oral administration of ASU in an in vivo sheep model of OA showed higher levels of PGs, a greater uncalcified cartilage thickness, and a significant reduction in subchondral bone sclerosis compared to control groups [98]. Interestingly, it has been shown that supplementation with ASU reduces the development of early OA cartilage and subchondral bone lesions in a canine model of OA with anterior cruciate ligament defects, and this effect appeared to be mediated by the inhibition of inducible nitric oxide synthase and MMP-13, which are key mediators of the structural changes that occur in OA [99]. Early ASU supplementation in mice has also been shown to stimulate the production of alkaline phosphatase, increase serum calcium levels, increase chondrocyte counts, and increase cartilage thickness in the middle part of the tibial plateau [100]. Furthermore, several studies have investigated TE approaches in which ASU was combined with biocompatible materials or scaffolds. Indeed, in a study with human adipose tissue-derived mesenchymal stem cells (hADSC) seeded on fibrin-alginate scaffolds, Hashemibeni and co-workers (2018) showed that Piascledin alone or in combination with TGF-β1 improves the proliferation, survival, and differentiation of hADSCs [91]. In contrast, in a study on soy isoflavones alone that examined the chondrogenic differentiation potential of hADSCs, no significantly higher increase in the expression of COL2A1 or COL2A1 could be found compared to the TGF-β1-stimulated control group [88]. 

Taken together, these results underline that a specific component in ASU alone may not have a large chondrogenic potential, but the combination of components in ASU can significantly enhance their anti-inflammatory effect and thus stimulate a suitable microenvironment for adequate progenitor cell chondrogenesis, thus leading to the repair and regeneration of cartilage lesions. 

## 6. Pomegranate

Pomegranate (*Punica granatum L.* (Punicaceae)) is a fruit and a rich source of two types of polyphenolic compounds: anthocyanins (such as dolphinidin, cyanidin, and pelargonidin), which give the fruit and juice its red color, and hydrolysable tannins (such as punicalin, pedunculagin, punicalagin, gallagic, and ellagic acid), which account for 92% of the anti-oxidant activity of the whole fruit [101,102]. More interestingly, studies have reported that the total anti-oxidant capacity of pomegranate juice is three times higher than that of popular anti-oxidant-containing beverages such as red wine and green tea, probably due to the presence of hydrolysable tannins in the peel together with anthocyanins and ellagic acid derivatives [103,104]. In a comparative analysis, it was found that anthocyanins from pomegranate have a higher anti-oxidant activity than vitamin E (a-tocopherol), ascorbic acid, or 3-carotene [103,105]. Pomegranate extract has been shown to protect against NSAIDs and ethanol-induced gastric ulcers [106], and the whole fruit and its extracts are used in many traditional medical systems to treat inflammation and pain in OA and other diseases [107]. It has been shown that the combination of phytochemicals found in the pomegranate fruit has a greater anti-inflammatory potential compared to a single ingredient application [108]. 

Previous studies have shown that a standardized pomegranate fruit extract (PFE) inhibits the production of MMPs by blocking the activation of p38-MAPK and master transcription factor NF-kB in OA chondrocytes [109], and they have also shown that the bioavailable metabolites of PFE inhibit the activity of COX-2 in OA chondrocytes [110]. Furthermore, it has also be shown that the pretreatment of human OA chondrocytes with PFE inhibits the interleukin IL-1β-induced activation of the upstream kinase MKK3 and the suppression of reactive oxygen species (ROS) levels, which lead to the inhibition of the p38 a-MAPK isoform and the activation and DNA-binding activity of the transcription factor RUNX-2 [111,112]. An in vivo study with rabbits showed that the consumption of PFE significantly reduced the expression of IL-6, MMPs, and PGE_2_, while the expression of AGC and COL2A1 was upregulated [113]. Furthermore, in vivo studies in mice have shown that the ingestion of PFE suppresses inflammation and joint destruction in a model of collagen-induced arthritis (CIA) [110], prevents the dose-dependent negative effects of iodoacetate [114], and exerts a significant chondroinductive potential on mouse embryos in vivo and limb bud cultures in vitro, with increased cell proliferation and differentiation rates [115]. In patients, the intake of PFE resulted in improved physical function, the reduced degradation of cartilage enzymes, and the increased anti-oxidant status in patients with knee OA [116]. Taken together, these data suggest that the consumption of PFE may be chondroprotective, chondroinductive, and a promising candidate for cartilage TE in the treatment of OA. 

## 7. Resveratrol 

The natural stilbenoid resveratrol (3,5,4′-trihydroxy-trans stilbene) is an active food-based ingredient found in more than 70 common plant species, including the skin of red grapes, blueberries, raspberries, mulberries, and peanuts. Though it has been used for centuries in traditional Chinese medicine in various herbal remedies, resveratrol itself was first isolated from the white hellebore (*Veratrum grandiflorum*) in 1939 and later from Japanese knotweed in 1963 [90,117,118]. Resveratrol has been shown to have a wide range of protective properties, including anti-oxidant, anti-inflammatory, anti-carcinogenic, cardio-protective, and immunomodulating properties [117,119,120]. The anti-inflammatory [34,121,122], anti-oxidant [123], anti-aging [124] and anti-OA properties [123,125], which are well-documented in the literature [126], are indeed remarkable. Furthermore, resveratrol has been reported to modulate the metabolism of lipids and to suppress the oxidation of low-density lipoproteins and the aggregation of platelets and other cells. Moreover, like phytoestrogen, resveratrol may provide cardiovascular protection, act in a chondroinductive fashion [127,128], and have a potential value for regulating bone resorption in age-related, hormone-dependent, and postmenopausal osteoporosis [129]. 

It is well-documented that pro-inflammatory cytokines such as IL-1β, TNF-α, and TNF-ß stimulate matrix-degrading enzymes such as MMPs and COX-2 by promoting NF-κB, which leads to the destruction of the cartilage matrix and joint inflammation and which plays an important role in the pathogenesis of RA and OA [37]. Moreover, it has been reported that COX-2 stimulation induces the synthesis of PGE_2_, which further mediates inflammation in tissues [130]. The traditional therapy of OA and RA is carried out by COX inhibitors (NSAIDs). However, NSAIDs have serious side effects such as stomach ulcers and do not block the synthesis of pro-inflammatory factors and proteins, which then further promote the breakdown of articular cartilage. This underlines the need for anti-inflammatory therapy, which on the one hand inhibits COX-2 (and thus prostaglandin synthesis) but on the other hand may modulate chondrocytes metabolism, further blocking progressive joint degeneration [131,132]. In fact, Subbaramaiah and co-workers demonstrated that resveratrol is a potential COX-2 inhibitor and the administration of resveratrol inhibited COX-2 expression and thus the production of PGE_2_ [133].

There is a plethora of studies that have explored the potential of resveratrol and OA treatment. In human chondrocytes, resveratrol has been shown to promote chondrocyte proliferation, the suppression of IL-1β-induced mitochondrial changes, blocking apoptosis, the upregulation of ROS, and the production of the tumor suppressor protein p53 [121]. In addition, resveratrol in combination with curcumin has shown synergistic effects on the suppression of NF-kB pathway activation and the activation of NF-κB-dependent gene end-products involved in inflammation (COX-2, MMP-9, and MMP-13), on the suppression of apoptosis by its inhibition of mitochondrial membrane depolarization and ATP depletion, and on the inhibition of caspase-3 activation [34,122,123,125,134]. Moreover, it was shown that the TNF-β induction of inflammatory pathways in primary human chondrocytes (PCH) can be modulated by resveratrol, and that down-regulation of histone deacetylase sirtuin-1 (SIRT1) by mRNA interference cancelled the effect of resveratrol on TNF-β-induced effects [58]. Furthermore, ultrastructural and cell viability studies have shown that resveratrol abolishes TNF-β-induced dose-dependent degrading/apoptotic morphological changes, cell viability, and proliferation in PCH [58]. Additionally, resveratrol significantly blocks the inflammatory-mediated suppression of the expression and synthesis of cartilage-specific ECM proteins (COL2A1 and AGC) and the cartilage-specific transcription factor SOX9 [125,135,136], and it inhibits the SNP-induced expression of p53 and p21 [135]. Additionally, in vitro investigations have demonstrated that pro-inflammatory cytokine effects, such as the IL-1β-induced inhibition of chondrocyte proliferation and morphological changes, are downregulated by resveratrol. Furthermore, resveratrol suppresses membrane-bound IL-1β and mature IL-1β protein synthesis in cartilage cells. Intriguingly, in IL-1β-stimulated cells, co-treatment with resveratrol down-regulates caspase-3, PARP cleavage, apoptosis, and the accumulation of the tumor suppressor gene p53, and it induces the ubiquitin-independent degradation of p53 [121]. Resveratrol has been further reported to down-regulate the IL-1β-promoted activation of pro-inflammatory transcription factor NF-kB and NF-κB-regulated pro-inflammatory and matrix-degrading gene biomarkers, including MMPs, caspase-3, VEGF, and COX-2. Furthermore, resveratrol suppresses IL-1β-induced IκBα degradation and, consequently, blocks IL-1β-induced IkBα phosphorylation. Thus, resveratrol suppresses the cytokine-promoted NF-κB-regulated expression of apoptosis-related gene products by stimulating the accumulation of phosphorylated IκBα, the ubiquitination of IκBα, and the inhibition of proteasome activity [34,37,122]. In addition, the in vivo effects of intra-articular injections of resveratrol on cartilage and synovium were investigated in a rabbit model of OA, in which resveratrol suppressed the degradation of cartilage tissue and had a chondroprotective effect on cartilage to prevent experimentally induced OA [137]. Resveratrol also proved to be a potent intracellular activator of the nuclear histone deacetylase sirtuin-1 (silent mating-type information regulation; SIRT1), which modulates the inflammatory effects of IL-1β, TNF-α, and TNF-β and suppresses the expression of HIF-2a [58,138]. Interestingly, it has been reported that the chondrogenic effects of resveratrol are at least partially mediated by the functional association between SIRT1 and SOX9, and interruption of this interaction leads to the inhibition of chondrogenesis [139]. However, the complex mechanism underlying resveratrol in chondrogenesis remains controversial, since resveratrol not only significantly upregulates SIRT1 gene expression but also puts chondrocytes into a hypertrophic state in vitro by upregulating COL1, COL10, and RUNX2 [124]. Furthermore, it was found that the chondroprotective effect of resveratrol is also related to chondrocyte autophagy by balancing HIF-1α and HIF-2α expressions and thus regulating the AMPK/mTOR signaling pathway [140]. In fact, it was reported that the administration of resveratrol in vivo significantly stimulated the activation of SIRT1 and the inhibition of HIF-2α expression in mouse OA cartilage tissue and in vitro in IL-1β-treated human chondrocytes. These data strongly indicate that the intra-articular injection of resveratrol significantly prevents the destruction of OA cartilage tissue through the stimulation of SIRT1, thus reducing the expression of HIF-2α and catabolic elements [138]. Recently, resveratrol has been found to exert anti-inflammatory activity in chondrocytes by upregulating miR-146b, thereby deactivating the NF-κB and p38MAPK signaling pathways [141]. The results of all these studies suggest that resveratrol actually acts as a multitargeting agent by modulating the inflammatory and apoptotic pathway in several steps. 

Since resveratrol offers such promising chondroprotective and inductive effects, several studies have investigated the effect of resveratrol in combination with three-dimensional (3D) environments on cartilage TE. The intra-articular injection of resveratrol at the beginning of OA protected cartilage from the development of experimentally induced OA in animal models [142]. Resveratrol promoted the chondrogenic differentiation of alginate-encapsulated MSCs by modulating β1-integrin signaling and blocking the IL-1β-mediated activation of NF-kB [117,143]. In 3D-alginate bead cultures and cartilage graft cultures, resveratrol improved the cell viability of articular chondrocytes, significantly increased BMP7-promoting PGs synthesis and suppressed the activation of transcription factors involved in inflammation and catabolic cartilage signaling pathways—including direct downstream regulators of MAPK and NF-κB and the deactivation of p53-induced apoptosis [134]. Loading a hyaluronic acid/hydrogel scaffold with resveratrol showed that the oxi-HA/resveratrol hydrogel is biocompatible with chondrocytes, enables ECM synthesis, and also reduces LPS-induced inflammation and damage [144]. In addition, Wang et al. (2014) incorporated a macromolecular drug consisting of resveratrol and polyacrylic acid into collagen to create anti-inflammatory cell-free scaffolds. The collagen/resveratrol scaffold had the ability to protect the chondrocytes from reactive oxygen species, osteochondral defects were completely repaired by the collagen/resveratrol scaffold, and the neocartilage integrated well with the surrounding tissue and subchondral bone [78]. Interestingly, a subsequent study showed that a continued release of resveratrol from the scaffold into the cell culture medium blocked IL-1β’s adverse effects (MMP-13 activation) and led to an upregulation of COL2A1, AGC, and SOX9 mRNA expression [145]. Taken together, results from many three-dimensional studies in vitro or in vivo underline the promising role of resveratrol for chondroprotective, chondroinductive, and cartilage TE in future applications. This active ingredient has stimulated innovative scientific concepts and raised public understanding of preventive health care.

## 8. Concluding Remarks

**Figure 2:**
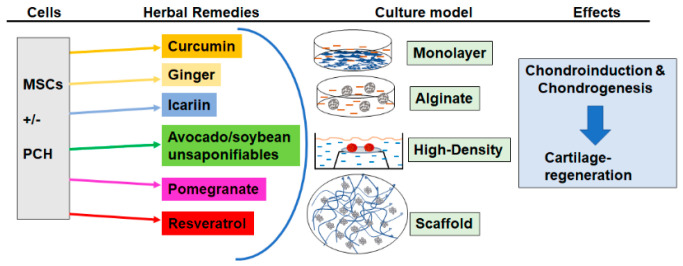
Schematic presentation of the application of plant phytochemicals in cartilage-TE.

The goal of this review was to evaluate the impact of plant-derived phytochemicals (herbal remedies) on cartilage TE and to highlight the chondrogenesis and chondroinductive effects of these compounds in studies on resident chondrocytes and progenitor cartilage cells (Figure 2). Articular cartilage defects are an increasingly frequent phenomenon in the aging community and cause severe pain, impaired joint function, and significant disability in OA patients. Plant-derived phytochemicals as potent anti-inflammatory and anti-oxidant agents are a very exciting and profound field of research, promising for their potential prophylactic properties and for the development of new therapeutic strategies for the treatment of pro-inflammatory diseases including OA. 

A large body of evidence has shown that a plethora of plants have solid pharmacological properties, including anti-inflammatory, anti-catabolic, and anti-apoptotic effects, and that they are capable to protect cartilage from the inflammatory and catabolic effects of various pro-inflammatory cytokines such as IL-1β, TNF-α, or TNF-β and destructive enzymes such as MMPs and inflammation mediators, COX-2, released by stimulated and inflamed synoviocytes and by inflammation-induced activated articular cartilage cells in the joints. Importantly, several studies have reported that the mentioned cytokines, pro-inflammatory, and degradation mediators are specifically regulated by the central master pro-inflammatory transcription factor NF-κB. Even more interesting is that the phytochemicals can effectively suppress the cytokine-induced degradation of the ECM in cartilage tissue by inhibiting the transcription factor NF-κB signaling pathway.

As discussed, and shown in this review, especially due to their anti-inflammatory and bactericidal properties, herbal remedies have vast potential in cartilage TE. Furthermore, an important aspect of successful cartilage TE is the maintenance of adequate scaffold microarchitecture and microenvironment so that chondrocytes or progenitor cells can maintain adequate chondrogenic morphology and promote chondrogenic matrix synthesis. This review highlights that herbal remedies are highly bio-compatible with various scaffold materials, and with constant drug release, they provide a favorable microenvironment supporting adequate cartilage repair mechanisms. 

To study the role of nutrients in cartilage as anabolic components in cartilage TE, prevention, and treatment in patients with OA, requires new strategies and large-scale clinical studies that are expected to take several decades. The clinical studies require a fundamental rethinking of the concept of clinical examinations in OA. The data should be clinically sound, and the number of exam patients across the population should be representative and the results should be reproducible.

It is also important that the tolerance levels (gastric tolerance, hepatotoxicity, kidney toxicity, and allergenicity) of the substances in patients is authorized by the European Food Safety Authority (EFSA) or the US Food and Drug Administration (FDA) [147,148]. Since the mechanisms of action of most herbal remedies are not yet fully understood and potential discrepancies could arise between the ingredients used in controlled clinical trials and those available to patients, the question of safety and tolerance as well as bioavailability does indeed arise. Therefore, for herbal medicinal products to be included in the routine treatment of OA, it is ultimately of great importance that pharmaceutical companies check and standardize the raw materials used to ensure the safety, efficacy, and quality of medicinal plant products. Here, the European Scientific Cooperative for Phytotherapy and the American Herbal Pharmacopoeia provide comprehensive information on animal experiments, clinical studies, the quality of medicinal plants, and recommendations for clinical use [149,150]. It is indeed important and expected that a broad acceptance of phytopharmaceutical interventions and dietary supplements by physicians is also required for OA patients with well-designed baseline, clinical, and epidemiological studies. Once these essential hurdles are overcome and answered, nutraceuticals (herbal remedies) can become a very helpful substitution and supplement to the pain relievers (e.g., NSAIDs), which are associated with many side effects and are very used commonly in large quantities worldwide to treat OA. 

Therefore, in the future, plant-derived phytochemicals, as promising non-toxic agents, anti-inflammatory, and anabolic compounds with chondroinductive potential, can be a beneficial complementary treatment for OA and regenerative cartilage TE approaches. 

## Figures and Tables

**Table 1:** Curcumin application in cartilage tissue engineering.

First Author	References	Experimental Model	Study Type	Findings
Shakibaei	[37]	The effects of curcumin were investigated in primary human chondrocytes (PCH) treated with interleukin-1 beta (IL-1β), tumor necrosis factor alpha (TNF-α). 90% collagen type II (COL2A1), integrin β1, cyclooxygenase-2 (COX-2), matrix metalloproteinase 9 (MMP-9), and the phosphorylation and nuclear translocation of NF-kB were analyzed.	in vitro/human chondrocytes	Curcumin as a naturally occurring anti-inflammatory agent for the treatment of osteoarthritis (OA) by suppressing the nuclear factor kappa B (NF-kB)-mediated IL-1β/TNF-α catabolic pathways in PCH.
Buhrmann	[48]	PCH from human samples treated with diverse cytokines, such as TNF-β, TNF-α, and/or curcumin in monolayer cultures.	in vitro/human chondrocytes	TNF-β is involved in the inflammatory microenvironment in PCH, similar to TNF-α, which leads to an upregulation of NF-κB signaling. The curcumin-inhibited action of cytokines can be used to maintain the chondrogenic potential of chondrocytes.
Shakibaei	[49]	PCH were pretreated with IL-1β, in monolayer and 3D-cultures with curcumin. The ultrastructure of PCH was investigated by TEM. The production of COL2A1, the β1 integrin, caspase–3 was analyzed by immunohistochemistry (IHC), Western immunoblotting (WB).	in vitro/human chondrocytes	Curcumin exerted anti-apoptotic, anti-catabolic effects on IL-1β-stimulated PCH. Curcumin has potential as an adjunct nutraceutical chondroprotective agent for treating OA and related osteoarticular disorders.
Wang	[50]	Chondrocytes were treated with IL-1β and co-treated with various concentrations of curcumin.	in vitro/rat chondrocytes	Curcumin inhibited the IL-1β-induced activation of NF-κB by suppressing pIκBα and p65/RelA nuclear translocation, suppressed MMP-13, and upregulated COL2A1 expression. Curcumin as anti-inflammatory agent for the treatment of OA by inhibition of NF-κB signaling.
Buhrmann	[53]	Mesenchymal stem cells (MSCs) were cultured in a ratio of 1:1 with primary chondrocytes in 3D-high-density culture with/without curcumin and/or IL-1β.	in vitro/chondrocytes, MSCs	Curcumin established a microenvironment, in which the effects of cytokines were antagonized, thus facilitating chondrogenesis of MSCs.
Perumal	[55]	Curcumin incorporated electrospun nanofibers of a blend of PLA and HPG for wound healing applications. Both the polymers are fabricated by electrospinning technique.	in vitro/curcumin-loaded biomaterials	(Poly(lactic acid)) (PLA)/hyperbranched polyglycerol (HPG)/curcumin nanofibers can be a potential wound patch dressing for acute and chronic wound applications.
Golchin	[56]	Nanofibrous scaffolds fabricated by chitosan (CS), PVA, carbopol, and polycaprolactone using a dual electrospinning technique while curcumin incorporated inside of the CS/PVA fibers.	in vitro/buccal fat pad-derived mesenchymal stem cells (BFP-MSCs)	This nanofibrous scaffold has great potential, as simultaneous administration of curcumin and BFP-MSCs holds the promising potential for use in various regenerative medicine applications.
Kim	[57]	Scaffold composed of curcumin and silk fibroin as an appropriate clinical replacement for defected cartilage. The scaffolds were designed to have adequate pore size and mechanical strength for cartilage repair.	in vitro/in vivo rat chondrocytes	The curcumin/silk scaffold showed its biocompatibility, a favorable environment for post-transplant cartilage repair in vivo. A functional composite scaffold of curcumin/silk can be used in cartilage-tissue engineering (TE) and as a promising substrate for cartilage repair.

**Table 2:** Ginger application in cartilage tissue engineering.

First Author	References	Experimental Model	Study Type	Findings
Bartels	[61]	Meta-analysis evaluating the clinical efficacy and safety of oral ginger consumption for the symptomatic treatment of OA.	in vivo/OA patients	Ginger lead to a statistically significant reduction in pain and OA symptoms compared to placebo control group and was well tolerated by the majority of OA patients.
Shen	[66,67]	Comparative effects of ginger on the synthesis of inflammatory mediators in normal and OA chondrocytes and cartilage explants	in vitro/chondrocytes, cartilage explants	Production of the pro-inflammatory mediators nitric oxide (NO) and prostaglandin E2 (PGE_2_) were significantly reduced with ginger extract in chondrocytes and cartilage explants.
Ruangsuriya	[68]	Zingerone was prepared in dimethyl sulphoxide (DMSO) and diluted to final concentrations in the culture media.	in vitro/chondrocytes	Zingerone suppressed the expression of TNF-α, IL-6, and IL-8 mRNA levels and reduced p38-mitogen-activated protein kinase (MAPK) and c-Jun *n*-terminal kinase phosphorylation, reducing cartilage inflammations and degradation.
Hosseinzadeh	[69]	Chondrocytes were pretreated with ginger extract and co-treated with IL-1β.	in vitro/chondrocytes	Ginger extract reduced IL-1β-induced oxidative stress, mitochondrial changes and apoptosis in chondrocytes.

**Table 3:** Icariin application in cartilage tissue engineering.

First Author	References	Experimental Model	Study Type	Findings
Zhang	[72]	Natural extracellular matrix (ECM)/PLLA scaffolds loading Ica (icariin), Ica–2-hydroxypropyl-β-cyclodextrin were prepared via phase separation, solvent replacement and freeze drying.	in vitro/chondrocytes	Ica–2-hydroxypropyl-β-cyclodextrin inclusion complex-loaded PLLA scaffolds are suitable for cartilage TE.
Wang	[73]	Ica was added to the chondrogenic medium in bone marrow MSCs cultures.	in vitro/chondrocytes, MSCs	Ica is an accelerant of growth factors for cartilage TE by promoting chondrogenesis of bone marrow MSCs but not hypertrophy.
Zhang	[74]	Rabbit chondrocytes were isolated and cultured with different concentrations of Ica.	in vitro/rabbit chondrocytes	Ica is an effective accelerant for chondrogenesis by up-regulation of the expression of aggrecan (AGC), COL2A1, and SOX9 genes. Ica-loaded biomaterials have the potential for cartilage TE.
Liu	[75]	Murine chondrocytes were treated with lipopolysaccharides (LPS) and co-cultured with various concentrations of Ica.	in vitro/mouse chondrocytes	Ica is a safe anabolic agent of chondrocytes, inhibits NO and MMP synthesis and may exert its protective effects by inhibition of NO and MMP synthesis, reduces the ECM destruction.
Kankala	[79]	Fabricated porous sodium alginate and gelatin 3D scaffold by 3D printing method. Cells were incubated with Ica.	in vitro/chondrocytes	Ica significantly enhanced the proliferation of chondrocytes, suggesting application for cartilage TE.
Yang, Li	[80,81]	Ica conjugated hyaluronic acid/collagen hydrogel were used for osteochondral defect repair.	in vitro/chondrocytes	Ica–hyaluronic acid (HA)/Col constructs facilitated reconstruction of osteochondral interface in rabbit subchondral defects. Ica–HA/Col hydrogel is a promising scaffold for restoring of osteochondral defect, suggesting application for cartilage TE.
Wang	[82]	Cells were seeded in plates, maintained in normal medium with Ica.	in vitro/in vivo chondrocytes	Ica treatment upregulated mRNA levels of SOX9, COL2A1, and AGC in the 3D cultures. Ica significantly enhanced cartilage repair.
Luo	[83]	Ica was administered into the chondrogenic medium for micromass culture of mouse chondrocytes.	in vitro/in vivo mouse chondrocytes	In mouse model of OA and chondrocytes in micromass cultures, Ica treatment reduced destruction of cartilage, promoted chondrocyte differentiation, upregulated expression of parathyroid hormone related proteins (PHrP), down-regulated the expression of Indian hedgehog (Ihh).
Liu, Zhang	[84,85]	Injectable thiolated Ica functionalized Col/HA hydrogel to promote cartilage formation. Ica-conditioned serum engineered with hyaluronic acid, articular cartilage defects in rabbit knees.	in vitro/in vivo cartilage	Ica–Col/HA hydrogel had great potential for clinical application in articular cartilage repair. Ica-conditioned serum combined with HA promotes reparative response in cartilage defects, the possible application in bioactive material-based cartilage regeneration therapies.

**Table 4:** Avocado/soybean unsaponifiables application in cartilage tissue engineering.

First Author	References	Experimental Model	Study Type	Findings
Henrotin	[88]	Chondrocytes cultured for 72 h with/without IL-1β and co-treated with avocado/soybean unsaponifiables (ASUs) were analyzed by HPLC and mass spectrometry. Anti-inflammatory and anabolic activity was investigated.	in vitro/human chondrocytes	ASU increased the expression of COL2A1 and AGC genes, cell proliferation. ASU partially reversed IL-1β effects on chondrocytes. Reduction of IL-1β effects were consistent with chondroprotective activity.
Boumediene	[93]	Articular chondrocytes were treated with various concentrations of ASU, the expression of transforming growth factor-β (TGF-β1),2, and their receptors (TGF-βRI and RII) was determined by Northern blot and PCR.	in vitro/bovine chondrocytes	ASU induced stimulation of ECM synthesis by the ability to enhance TGF-β expression. ASU increased the production of plasminogen activator inhibitor (PAI-1), lead to inhibition of MMP, and induced matrix repair mechanisms in chondrocytes.
Frondoza	[95]	Evaluation of the effects of ASU/α-lipoic acid (LA) on production of PGE_2_ in equine chondrocyte stimulated with LPS, IL-1β, or H_2_O_2_ for 24 h, and supernatants were immunoassayed for PGE_2_.	in vitro/equine chondrocytes	Chondrocyte PGE_2_ production was inhibited by ASU/LA more effectively than either alone, which was associated with the suppression of NF-κB translocation. ASU/LA on PGE_2_ production has a potential for anti-inflammatory/antioxidant approach in OA.
Au	[96]	Evaluation of ASU on pro-inflammatory mediators in chondrocytes and monocyte/ macrophage-like cells. Cells were stimulated for 1 h with LPS, and analyzed for TNF-α, IL-1β, COX-2, inducible nitric oxide synthase (iNOS), and PGE_2_ expression.	in vitro/chondrocytes	ASU reduced TNF-α, IL-1β, COX-2, PGE_2_, and iNOS expression in LPS-stimulated chondrocytes, and anti-inflammatory effects of ASU were observed. ASU also reduced TNF-α and IL-1β expression in LPS-stimulated monocyte/ macrophage-like cells.
Lippiello	[97]	ASU samples were analyzed by HPLC, gas chromatography (GC) mass spectrometry, to clarify, if the sterol of ASU were the primary contributors to biological activity in chondrocytes. The sterol content was normalized between diverse samples prior to testing on chondrocytes. Anti-inflammatory activity was assayed by measuring of IL-1β-induced synthesis of PGE_2_, MMPs, release of S-35.	in vitro/chondrocytes	ASU samples exerted a time-dependent upregulation of the 35-sulphate uptake in chondrocytes. ASU were effective in the dose-dependent inhibition of IL-1β-induced MMP-3 activity, PGE_2_ synthesis. The upregulation of glycosaminoglycans (GAGs), collagen synthesis, and the reduction of IL-1β effects in cartilage were compatible with chondroprotective activity.
Hashemibeni	[91]	Isolated human adipose tissue-derived mesenchymal stem cells (hADSCs) were seeded in fibrin or fibrin-alginate scaffolds in chondrogenic medium containing Piascledin, TGF-β1, or both.	in vitro/hADSCs/chondrocytes	Piascledin was able to enhance the proliferation, survival of hADSCs in scaffolds. However, the expression of COL2A1 was higher in the TGF-β1 groups, and the expression of AGC was higher in TGF-β1 alone or with Piascledin in scaffolds.

**Table 5:** Pomegranate application in cartilage tissue engineering.

First Author	References	Experimental Model	Study Type	Findings
Ahmed	[109]	OA chondrocytes or cartilage explants were pre-treated with pomegranate fruit extract (PFE), co-treated with IL-1β. The amounts of PG were measured with a colorimetric assay. The expression of MMPs, pIkBα, and MAPKs was determined by WB and NF-kB by electrophoretic mobility shift assay (EMSA).	in vitro/cartilage explants, chondrocytes	PFE inhibited the IL-1β-induced PG breakdown, MMPs expression on protein and mRNA level, p38-MAPK, phosphorylation of inhibitor of kappa B alpha (IkBα), and NF-kB binding to DNA in OA cartilage explants.
Haseeb	[111]	The potential of PFE to suppress IL-1β-stimulated expression of IL-6, reactive oxygen species (ROS), and NF-κB by analyzing the activation of the kinases upstream of IκBα in PCH by WB.	in vitro/human chondrocytes	PFE inhibited the mRNA and protein expression of IL-6, ROS, and IL-1β-mediated phosphorylation IKKβ, degradation of IκBα, and activation and nuclear translocation of NF-κB/p65 in human chondrocytes. PFE exerted chondroprotective effects by suppressing the NF-kB pathway.
Akhtar	[113]	OA was surgically induced in the tibiofemoral joints of rabbits. In one group, animals were fed PFE in water for 8 wks postsurgery. In the second group, animals were fed PFE for 2 wks before surgery and for 8 wks postsurgery.	in vitro/in vivo rabbit chondrocytes	PFE-fed rabbits had lower levels of IL-6, MMP-13, and PGE_2_ in synovial fluid/plasma and showed higher expression of AGC and COL2A1 mRNA. PFE treatment significantly reduced IL-1β-induced MAPK and NF-κB inhibitors, and PGE_2_ production, which highlighted the chondroprotective effect of PFE in the treatment of OA.
Monsefi	[115]	Pregnant BALB/c mice were given PFE to investigate the effect on chondrogenesis. Their embryos were stained with alizarin red S and alcian blue. Bone Ca content in pregnant mice was also measured.	in vitro/in vivo mouse chondrocytes, MSCs	PFE was able to enhance bone/ cartilage formation. MSCs from fetal limb buds were cultured, exposed to PFE, the number of viable cells was greater than in control cultures. The number of cartilage nodules and their diameters were greater in PFE-treated cultures.
Ghoochani	[116]	Patients with knee OA and control drank 200 mL PFE/daily for 6 weeks, and the effect of this intervention on clinical signs was evaluated.	in vivo/OA patients	Significant increases in physical function of decrease in breakdown of cartilage enzymes and increase of anti-oxidant status in patients with knee OA were observed in PFE group.

**Table 6:** Resveratrol application in cartilage tissue engineering.

First Author	References	Experimental Model	Study Type	Findings
Csaki, Shakibaei	[34,146]	Investigation of the potential synergistic effects of resveratrol or/and curcumin on IL-1β-stimulated human PCH using WB and electron microscopy (EM).	in vitro/human chondrocytes	Both compounds targeted the NF-κB and MAPK pathways. Resveratrol inhibits the proteasome, while curcumin modulates inhibiting upstream kinases and MAPK.
Buhrmann	[58]	PCH were cultured in 3D-alginate cultures, resveratrol was prepared in ethanol, diluted in medium. Alginate cultures of PCH were treated with TNF-β, TNF-α, or T-lymphocytes and co-treated with resveratrol.	in vitro/human chondrocytes	Suppression of TNF-β-, similar to TNF-α- or T-lymphocytes-induced inflammatory microenvironment in PCH by resveratrol/histone deacetylase sirtuin-1 (SIRT1), might be a novel therapeutic approach for targeting inflammation during OA/RA.
Wang	[78]	Resveratrol grafted to polyacrylic acid to fabricate collagen/resveratrol scaffolds and chondrocytes seeded on the scaffolds.	in vitro/in vivo chondrocytes	Osteochondral defects were completely repaired by the collagen/resveratrol scaffold, and the neo-cartilage integrated well with its surrounding tissue.
Csaki, Shakibaei	[121,122]	Human PCH in monolayer cultures treated with IL-1β and co-treated with various concentrations of resveratrol and evaluated with ((3-(4,5-dimethylthiazol-2-yl)-2,5-diphenyl-tetrazolium-bromid)) (MTT) assay, WB, and EM.	in vitro/human chondrocytes	Resveratrol inhibited the expression of VEGF, MMP-3, MMP-9, and COX-2 in PCH stimulated with IL-1β. Resveratrol exerted a chondroprotective capacity by suppression of IL-1β, ROS, p53-production. and apoptosis by down-regulation of NF-kB.
Dave	[123]	Human chondrocytes and cartilage explants were isolated from OA patients, treated with IL-1β and/or resveratrol. Evaluation of PGE_2_, leukotriene B (LTB4), COX, and MMP expression, as well as PG production.	in vitro/in vivo chondrocytes	Resveratrol inhibited chondrocyte apoptosis via inhibition of COX-2-derived PGE_2_ synthesis by suppression of mitochondrial membrane depolarization, depletion. Resveratrol protected against oxidant injury and apoptosis, which are main features of progressive OA.
Kim	[124]	Healthy and OA chondrocytes were incubated with various concentrations of resveratrol. Cell proliferation and gene expressions were evaluated.	in vitro/chondrocytes	Resveratrol treatment significantly upregulated SIRT1 gene expression in normal and OA chondrocytes.
Liu	[125]	Chondrocytes were isolated from pig joints; Resveratrol was prepared as a stock solution with ethanol. Activation of the IκBα, NF-kB, and MAPK, activator protein-1 (AP-1) pathways was assessed by EMSA, WB, and transfection assay.	in vitro/in vivo porcine chondrocytes	iNOS, COX-2, PGE_2_ were suppressed by resveratrol, mediated by inhibiting IKK-IκBα-NF-κB and JNK/ERK/AP-1pathways induced by advanced glycation end products (AGEs). Resveratrol could prevent AGEs-induced degradation of PG and AGC in cartilage explants.
Im	[134]	Isolated human PCH, cultured in monolayer or in 3D-alginate cultures and treated with resveratrol.	in vitro/human chondrocytes	Resveratrol improved the viability of PCH, antagonized protease production, and promoted PG synthesis as evaluated by (35) S-sulfate incorporation. Modulation of the downstream regulators of MAPK and NF-κB. Resveratrol exerted its chondroprotective actions partly by inhibition p53-induced apoptosis but not in chondrosarcoma.
Eo	[135]	The effect of resveratrol on NO-induced apoptosis in rabbit articular chondrocytes was investigated.	in vitro/chondrocytes	Resveratrol inhibited NO-induced apoptosis through the NF-κB signaling pathway in articular chondrocytes.
Maepa	[136]	Porcine articular chondrocytes were isolated cultured as monolayers, treated with resveratrol.	in vitro/porcine chondrocytes	Resveratrol stimulated the expression of collagen II, regulated collagen II protein in different zones of articular cartilage.
Elmali	[137]	OA model, a unilateral anterior cruciate ligament transection (ACLT) was performed in rabbits. Five weeks after the test group had been injected daily with 10 μM/kg resveratrol in DMSO into the knees for two weeks, the control group was injected DMSO into the knees.	in vivo/rabbit chondrocytes	Intraarticular injections of resveratrol starting at the onset of OA disease may protect cartilage against the development of experimentally induced OA.
Li	[138]	Intra-articular injection of resveratrol into a mouse model of OA. OA was induced in the mouse knee using the destabilization of the medial meniscus (DMM). Resveratrol was injected weekly into the operated knee starting 4 weeks after surgery. The OA phenotype was investigated by histological and IHC analyses.	in vitro/in vivo human chondrocytes	Injection of resveratrol significantly prevented the destruction of OA cartilage by stimulating SIRT1, thereby suppressing the expression of HIF-2α and catabolic factors. Activation of SIRT1, the inhibition of HIF-2α in cartilage tissue and in vitro in IL-1β-treated chondrocytes.
Jin	[141]	Mouse chondrogenic cells were treated with 30 μM resveratrol for 24 h and 10 μg/mL LPS for 12 h. Cell viability, apoptosis and the release of pro-inflammatory cytokines was assessed.	in vitro/mouse chondrocytes	Resveratrol supported chondrogenic cell line of LPS-induced inflammatory apoptotic effects by upregulation of miR-146b, and deactivation of NF-κB, p38-MAPK signaling.
Lei	[143]	MSC-derived chondrocytes cultured on CGS, treated with IL-1β and co-treated with resveratrol and evaluated ECM, MMPs, and NF-kB.	in vitro/MSCs chondrocytes	Resveratrol acted as a NF-kB inhibitor to protect MSC-derived chondrocytes on the CGS from IL-1β catabolism and these effects were mediated by β1-integrin.
Sheu	[144]	Fabricated and characterized an Oxi-HA/resveratrol hydrogel for future applications in cartilage TE.	in vitro/chondrocytes	Oxi-HA/resveratrol hydrogel up-regulated COL2A1, AGC, and SOX-9 genes, down-regulated IL-1β and MMPs genes. Oxi-HA/resveratrol hydrogel is biocompatible with chondrocytes, allows ECM synthesis, a potentially suitable cell carrier for chondrocyte in the treatment of cartilage defects
Wu	[145]	Resveratrol-loaded microspheres were fabricated using oil-in-water emulsion and solution-evaporation methods. Human bone marrow MSCs were treated with IL-1β and co-treated with resveratrol.	in vitro/hMSCs	Resveratrol inhibited the activity of IL-1β, thereby downregulating MMP-13 mRNA expression. Up-regulation of COL2A1, AGC, and Sox9 mRNA expression. Resveratrol maintained chondrogenic gene expression of cells when exposed to the inflammatory agents.

## Abbreviations

3D	Three-Dimensional
ACI	Autologous Chondrocyte Implantation
ACLT	Anterior Cruciate Ligament Transection
AGC	Aggrecan
AGEs	Advanced Glycation End products
AMPK/mTOR	Adenosine Monophosphate-activated Protein Kinase/rapamycin
AP-1	Activator Protein-1
ASU	Avocado/Soybean Unsaponifiable (ASU)
BALB/c	Bagg Albino Laboratory-Bred strain c of the house mouse
BFP-MSCs	Buccal Fat Pad-Derived Mesenchymal Stem Cells
BMP-6	Bone Morphogenic Protein-6
CIA	Collagen-Induced Arthritis
COL2A1	Collagen type II
COX-2	Cyclooxygenase-2
CS	Chitosan
CSG	Chitosan-Gelatin Scaffolds
CSPGs	Cartilage-Specific Proteoglycans
DJD	Degenerative Joint Disease
DMEM	Dulbecco’s Modified Eagle’s Medium
DMM	Destabilization of the Medial Meniscus
DMSO	Dimethyl Sulphoxide
ECM	Extracellular Matrix
EFSA	European Food Safety Authority
EM	Electron Microscopy
EMSA	Electrophoretic Mobility Shift Assay
FDA	US Food and Drug Administration
GA	Glutaraldehyde
GAGs	Glycosaminoglycans
GC	Gas Chromatography
Gel/HA	Gelatin/Hyaluronic Acid
GlcN	Glucosamine
GRE	Ginger Root Extract
H2O2	Hydrogen peroxide
hADSCs	Human Adipose Tissue-Derived Mesenchymal Stem Cells
HIF	Hypoxia-Inducible Factor
HPG	Hyperbranched Polyglycerol
HPLS	High Pressure Liquid Chromatography
Ica	Icariin
IGF-1	Insulin-Like Growth Factor-1
IHC	Immunohistochemistry
Ihh	Indian Hedgehog
IkBα	Inhibitor of Kappa B Alpha
IKKβ	inhibitor of Nuclear Factor Kappa-B Kinase Subunit Beta (IKKβ),
IL-1β	Interleukin-1β
IGFBP-3	Insulin-like-growth-factor-binding-protein-3
iNOS	Inducible Nitric Oxide Synthase
JNK/ERK/AP-1	c-Jun N-terminal kinase/Extracellular signal-Regulated Kinase/Activator Protein -1 pathways
LA	α-Lipoic Acid
LPS	Lipopolysaccharides
LTB	Leukotriene B
MAPK	Mitogen-Activated Protein Kinase
MKK3	Mitogen-activated protein kinase kinase 3
MMP	Matrix Metalloproteinase
MSCs	Mesenchymal Stem Cells
MTT	((3-(4,5-Dimethylthiazol-2-yl)-2,5-Diphenyl-Tetrazolium-Bromid))
NF-κB	Nuclear Factor-Kappa B
NO	Nitric Oxide
NSAIDs	Non-Steroidal Anti-Inflammatory Drugs
OA	Osteoarthritis
Oxi-HA/Res	Oxidized Hyaluronic Acid/Resveratrol
PAI	Plasminogen Activator Inhibitor
PARP	Poly(ADP-ribose)-Polymerasen
PCH	Primary Human Chondrocytes
PCR	Polymerase Chain Reaction.
PFE	Pomegranate Fruit Extract
PG	Proteoglycan
PGE_2_	Prostaglandin E2
PHrP	Parathyroid Hormone-Related Proteins
PLA	Poly(Lactic Acid)
PLLA	Poly(L-lactic acid)
PVA	Poly(Vinyl Alcohol)
RA	Rheumatoid Arthritis
ROS	Reactive Oxygen Species
**RUNX-2**	**Runt-related transcription factor 2**
S-35	35-sulfate into proteoglycans
SIRT1	Histone Deacetylase Sirtuin-1
Sirtuin-1	Silent Mating-Type Information Regulation-1
SNP	sodium nitroprusside
SOX9	(Sex-Determining Region Y)-Box
TE	Tissue Engineering
TGF-β	Transforming Growth Factor-β
TGF-βRI	Transforming growth factor beta receptor I
TNF-α	Tumor-Necrosis Factor
VEGF	Vascular Endothelial Growth Factor
WB	Western Immunoblotting

## References

[1] Aigner T., Rose J., Martin J., Buckwalter J. (2004). Aging theories of primary osteoarthritis: From epidemiology to molecular biology. Rejuvenation Res..

[2] Lawrence R.C., Felson D.T., Helmick C.G., Arnold L.M., Choi H., Deyo R.A., Gabriel S., Hirsch R., Hochberg M.C., Hunder G.G. (2008). Estimates of the prevalence of arthritis and other rheumatic conditions in the United States. Part II. Arthritis Rheum..

[3] Cushnaghan J., Dieppe P. (1991). Study of 500 patients with limb joint osteoarthritis. I. Analysis by age, sex, and distribution of symptomatic joint sites. Ann. Rheum. Dis..

[4] O’Neill T.W., McCabe P.S., McBeth J. (2018). Update on the epidemiology, risk factors and disease outcomes of osteoarthritis. Best Pr. Res. Clin. Haematol..

[5] Mobasheri A. (2012). Intersection of inflammation and herbal medicine in the treatment of osteoarthritis. Curr. Rheumatol. Rep..

[6] Roach H.I., Aigner T., Soder S., Haag J., Welkerling H. (2007). Pathobiology of osteoarthritis: Pathomechanisms and potential therapeutic targets. Curr. Drug Targets.

[7] Goldring M.B., Goldring S.R. (2007). Osteoarthritis. J. Cell. Physiol..

[8] Sutton S., Clutterbuck A., Harris P., Gent T., Freeman S., Foster N., Barrett-Jolley R., Mobasheri A. (2009). The contribution of the synovium, synovial derived inflammatory cytokines and neuropeptides to the pathogenesis of osteoarthritis. Vet. J. (Lond. Engl. 1997).

[9] Ilyin S.E., Belkowski S.M., Plata-Salamán C.R. (2004). Biomarker discovery and validation: Technologies and integrative approaches. Trends Biotechnol..

[10] Buckwalter J.A., Mankin H.J. (1998). Articular cartilage: Degeneration and osteoarthritis, repair, regeneration, and transplantation. Instr. Course Lect..

[11] Mobasheri A., Airley R., Foster C.S., Schulze-Tanzil G., Shakibaei M. (2004). Post-genomic applications of tissue microarrays: Basic research, prognostic oncology, clinical genomics and drug discovery. Histol. Histopathol..

[12] Dieppe P.A., Lohmander L.S. (2005). Pathogenesis and management of pain in osteoarthritis. Lancet (Lond. Engl.).

[13] Messina O.D., Vidal Wilman M., Vidal Neira L.F. (2019). Nutrition, osteoarthritis and cartilage metabolism. Aging Clin. Exp. Res..

[14] Shakibaei M., Allaway D., Nebrich S., Mobasheri A. (2012). Botanical extracts from rosehip (Rosa canina), willow bark (Salix alba), and nettle leaf (Urtica dioica) suppress IL-1β-Induced NF-κB activation in canine articular chondrocytes. Evid. -Based Complementary Altern. Med. Ecam.

[15] Cancedda R., Dozin B., Giannoni P., Quarto R. (2003). Tissue engineering and cell therapy of cartilage and bone. Matrix Biol. J. Int. Soc. Matrix Biol..

[16] Vachon A., Bramlage L.R., Gabel A.A., Weisbrode S. (1986). Evaluation of the repair process of cartilage defects of the equine third carpal bone with and without subchondral bone perforation. Am. J. Vet. Res..

[17] Kuettner K.E. (1992). Biochemistry of articular cartilage in health and disease. Clin. Biochem..

[18] Qi W.N., Scully S.P. (2003). Type II collagen modulates the composition of extracellular matrix synthesized by articular chondrocytes. J. Orthop. Res.: Off. Publ. Orthop. Res. Soc..

[19] Mobasheri A., Csaki C., Clutterbuck A.L., Rahmanzadeh M., Shakibaei M. (2009). Mesenchymal stem cells in connective tissue engineering and regenerative medicine: Applications in cartilage repair and osteoarthritis therapy. Histol. Histopathol..

[20] Csaki C., Schneider P.R., Shakibaei M. (2008). Mesenchymal stem cells as a potential pool for cartilage tissue engineering. Ann. Anat. Anat. Anz. Off. Organ Anat. Ges..

[21] Huselstein C., Li Y., He X. (2012). Mesenchymal stem cells for cartilage engineering. Bio-Med. Mater. Eng..

[22] Chen D., Zhao M., Mundy G.R. (2004). Bone morphogenetic proteins. Growth Factors (ChurSwitz.).

[23] Kabiri A., Esfandiari E., Hashemibeni B., Kazemi M., Mardani M., Esmaeili A. (2012). Effects of FGF-2 on human adipose tissue derived adult stem cells morphology and chondrogenesis enhancement in Transwell culture. Biochem. Biophys. Res. Commun..

[24] Martin J.A., Scherb M.B., Lembke L.A., Buckwalter J.A. (2000). Damage control mechanisms in articular cartilage: The role of the insulin-like growth factor I axis. Iowa Orthop. J..

[25] Hutmacher D.W. (2000). Scaffolds in tissue engineering bone and cartilage. Biomaterials.

[26] Loeser R.F. (1997). Growth factor regulation of chondrocyte integrins. Differential effects of insulin-like growth factor 1 and transforming growth factor beta on alpha 1 beta 1 integrin expression and chondrocyte adhesion to type VI collagen. Arthritis Rheum..

[27] Hollander A.P., Dickinson S.C., Sims T.J., Brun P., Cortivo R., Kon E., Marcacci M., Zanasi S., Borrione A., De Luca C. (2006). Maturation of tissue engineered cartilage implanted in injured and osteoarthritic human knees. Tissue Eng..

[28] Caplan A.I., Goldberg V.M. (1999). Principles of tissue engineered regeneration of skeletal tissues. Clin. Orthop. Relat. Res..

[29] Solchaga L.A., Goldberg V.M., Caplan A.I. (2001). Cartilage regeneration using principles of tissue engineering. Clin. Orthop. Relat. Res..

[30] Armiento A.R., Stoddart M.J., Alini M., Eglin D. (2018). Biomaterials for articular cartilage tissue engineering: Learning from biology. Acta Biomater..

[31] Caplan A.I. (2007). Adult mesenchymal stem cells for tissue engineering versus regenerative medicine. J. Cell. Physiol..

[32] Grevenstein D., Mamilos A., Schmitt V.H., Niedermair T., Wagner W., Kirkpatrick C.J., Brochhausen C. (2020). Excellent histological results in terms of articular cartilage regeneration after spheroid-based autologous chondrocyte implantation (ACI). Knee Surg. Sports Traumatol. Arthrosc. Off. J. Esska.

[33] Sun M., Lu Z., Cai P., Zheng L., Zhao J. (2020). Salidroside enhances proliferation and maintains phenotype of articular chondrocytes for autologous chondrocyte implantation (ACI) via TGF-beta/Smad3 Signal. Biomed. Pharm. Biomed. Pharmacother..

[34] Csaki C., Mobasheri A., Shakibaei M. (2009). Synergistic chondroprotective effects of curcumin and resveratrol in human articular chondrocytes: Inhibition of IL-1beta-induced NF-kappaB-mediated inflammation and apoptosis. Arthritis Res. Ther..

[35] Mobasheri A., Henrotin Y., Biesalski H.K., Shakibaei M. (2012). Scientific evidence and rationale for the development of curcumin and resveratrol as nutraceutricals for joint health. Int. J. Mol. Sci..

[36] Schulze-Tanzil G., Mobasheri A., Sendzik J., John T., Shakibaei M. (2004). Effects of curcumin (diferuloylmethane) on nuclear factor kappaB signaling in interleukin-1beta-stimulated chondrocytes. Ann. N.Y. Acad. Sci..

[37] Shakibaei M., John T., Schulze-Tanzil G., Lehmann I., Mobasheri A. (2007). Suppression of NF-kappaB activation by curcumin leads to inhibition of expression of cyclo-oxygenase-2 and matrix metalloproteinase-9 in human articular chondrocytes: Implications for the treatment of osteoarthritis. Biochem. Pharm..

[38] Ameye L.G., Chee W.S. (2006). Osteoarthritis and nutrition. From nutraceuticals to functional foods: A systematic review of the scientific evidence. Arthritis Res. Ther..

[39] Henrotin Y., Lambert C., Couchourel D., Ripoll C., Chiotelli E. (2011). Nutraceuticals: Do they represent a new era in the management of osteoarthritis? - a narrative review from the lessons taken with five products. Osteoarthr. Cartil..

[40] Kalra E.K. (2003). Nutraceutical-definition and introduction. Aaps Pharmsci.

[41] Hak A.E., Choi H.K. (2008). Lifestyle and gout. Curr. Opin. Rheumatol..

[42] Sale J.E., Gignac M., Hawker G. (2008). The relationship between disease symptoms, life events, coping and treatment, and depression among older adults with osteoarthritis. J. Rheumatol..

[43] Henrotin Y., Clutterbuck A.L., Allaway D., Lodwig E.M., Harris P., Mathy-Hartert M., Shakibaei M., Mobasheri A. (2010). Biological actions of curcumin on articular chondrocytes. Osteoarthr. Cartil..

[44] Schulze-Tanzil G., de Souza P.H., Behnke B., Klingelhoefer S., Scheid A., Shakibaei M. (2002). Effects of the antirheumatic remedy hox alpha-a new stinging nettle leaf extract-on matrix metalloproteinases in human chondrocytes in vitro. Histol. Histopathol..

[45] Shen C.L., Smith B.J., Lo D.F., Chyu M.C., Dunn D.M., Chen C.H., Kwun I.S. (2012). Dietary polyphenols and mechanisms of osteoarthritis. Nutr. Biochem..

[46] Long L., Soeken K., Ernst E. (2001). Herbal medicines for the treatment of osteoarthritis: A systematic review. Rheumatology (Oxf. Engl.).

[47] Kunnumakkara A.B., Bordoloi D., Padmavathi G., Monisha J., Roy N.K., Prasad S., Aggarwal B.B. (2017). Curcumin, the golden nutraceutical: Multitargeting for multiple chronic diseases. Br. J. Pharm...

[48] Buhrmann C., Shayan P., Aggarwal B.B., Shakibaei M. (2013). Evidence that TNF-beta (lymphotoxin alpha) can activate the inflammatory environment in human chondrocytes. Arthritis Res. Ther..

[49] Shakibaei M., Schulze-Tanzil G., John T., Mobasheri A. (2005). Curcumin protects human chondrocytes from IL-l1beta-induced inhibition of collagen type II and beta1-integrin expression and activation of caspase-3: An immunomorphological study. Ann. Anat. Anat. Anz. Off. Organ Anat. Ges..

[50] Wang J., Ma J., Gu J.H., Wang F.Y., Shang X.S., Tao H.R., Wang X. (2017). Regulation of type II collagen, matrix metalloproteinase-13 and cell proliferation by interleukin-1beta is mediated by curcumin via inhibition of NF-kappaB signaling in rat chondrocytes. Mol. Med. Rep..

[51] Clutterbuck A.L., Mobasheri A., Shakibaei M., Allaway D., Harris P. (2009). Interleukin-1β–Induced extracellular matrix degradation and glycosaminoglycan release is inhibited by curcumin in an explant model of cartilage inflammation. Ann. N.Y. Acad. Sci..

[52] Toegel S., Wu S.Q., Piana C., Unger F.M., Wirth M., Goldring M.B., Gabor F., Viernstein H. (2008). Comparison between chondroprotective effects of glucosamine, curcumin, and diacerein in IL-1beta-stimulated C-28/I2 chondrocytes. Osteoarthr. Cartil..

[53] Buhrmann C., Mobasheri A., Matis U., Shakibaei M. (2010). Curcumin mediated suppression of nuclear factor-kappaB promotes chondrogenic differentiation of mesenchymal stem cells in a high-density co-culture microenvironment. Arthritis Res. Ther..

[54] Chen Y., Lin J., Fei Y., Wang H., Gao W. (2010). Preparation and characterization of electrospinning PLA/curcumin composite membranes. Fibers Polym..

[55] Perumal G., Pappuru S., Chakraborty D., Maya Nandkumar A., Chand D.K., Doble M. (2017). Synthesis and characterization of curcumin loaded PLA-Hyperbranched polyglycerol electrospun blend for wound dressing applications. Mater. Sci. Eng. C Mater. Biol. Appl..

[56] Golchin A., Hosseinzadeh S., Staji M., Soleimani M., Ardeshirylajimi A., Khojasteh A. (2019). Biological behavior of the curcumin incorporated chitosan/poly(vinyl alcohol) nanofibers for biomedical applications. J. Cell. Biochem..

[57] Kim D.K., In Kim J., Sim B.R., Khang G. (2017). Bioengineered porous composite curcumin/silk scaffolds for cartilage regeneration. Mater. Sci. Eng. C Mater. Biol. Appl..

[58] Buhrmann C., Popper B., Aggarwal B.B., Shakibaei M. (2017). Resveratrol downregulates inflammatory pathway activated by lymphotoxin alpha (TNF-beta) in articular chondrocytes: Comparison with TNF-alpha. PLoS ONE.

[59] Ali B.H., Blunden G., Tanira M.O., Nemmar A. (2008). Some phytochemical, pharmacological and toxicological properties of ginger (Zingiber officinale Roscoe): A review of recent research. Food Chem. Toxicol. Int. J. Publ. Br. Ind. Biol. Res. Assoc..

[60] Tjendraputra E., Tran V.H., Liu-Brennan D., Roufogalis B.D., Duke C.C. (2001). Effect of ginger constituents and synthetic analogues on cyclooxygenase-2 enzyme in intact cells. Bioorg. Chem..

[61] Bartels E.M., Folmer V.N., Bliddal H., Altman R.D., Juhl C., Tarp S., Zhang W., Christensen R. (2015). Efficacy and safety of ginger in osteoarthritis patients: A meta-analysis of randomized placebo-controlled trials. Osteoarthr. Cartil..

[62] Grzanna R., Lindmark L., Frondoza C.G. (2005). Ginger-an herbal medicinal product with broad anti-inflammatory actions. J. Med. Food.

[63] van Breemen R.B., Tao Y., Li W. (2011). Cyclooxygenase-2 inhibitors in ginger (Zingiber officinale). Fitoterapia.

[64] Kiuchi F., Iwakami S., Shibuya M., Hanaoka F., Sankawa U. (1992). Inhibition of prostaglandin and leukotriene biosynthesis by gingerols and diarylheptanoids. Chem. Pharm. Bull..

[65] Phan P.V., Sohrabi A., Polotsky A., Hungerford D.S., Lindmark L., Frondoza C.G. (2005). Ginger extract components suppress induction of chemokine expression in human synoviocytes. J. Altern. Complementary Med. (N.Y.).

[66] Shen C.L., Hong K.J., Kim S.W. (2003). Effects of ginger (Zingiber officinale Rosc.) on decreasing the production of inflammatory mediators in sow osteoarthrotic cartilage explants. J. Med. Food.

[67] Shen C.L., Hong K.J., Kim S.W. (2005). Comparative effects of ginger root (Zingiber officinale Rosc.) on the production of inflammatory mediators in normal and osteoarthrotic sow chondrocytes. J. Med. Food.

[68] Ruangsuriya J., Budprom P., Viriyakhasem N., Kongdang P., Chokchaitaweesuk C., Sirikaew N., Chomdej S., Nganvongpanit K., Ongchai S. (2017). Suppression of cartilage degradation by zingerone involving the p38 and JNK MAPK signaling pathway. Planta Med..

[69] Hosseinzadeh A., Bahrampour Juybari K., Fatemi M.J., Kamarul T., Bagheri A., Tekiyehmaroof N., Sharifi A.M. (2017). Protective effect of ginger (Zingiber officinale Roscoe) extract against oxidative stress and mitochondrial apoptosis induced by interleukin-1β in cultured chondrocytes. Cells Tissues Organs.

[70] Ming L.G., Chen K.M., Xian C.J. (2013). Functions and action mechanisms of flavonoids genistein and icariin in regulating bone remodeling. J. Cell. Physiol..

[71] Mi B., Wang J., Liu Y., Liu J., Hu L., Panayi A.C., Liu G., Zhou W. (2018). Icariin activates autophagy via Down-Regulation of the NF-κB Signaling-Mediated apoptosis in chondrocytes. Front. Pharm..

[72] Zhang X., Liu T., Huang Y., Wismeijer D., Liu Y. (2014). Icariin: Does it have an osteoinductive potential for bone tissue engineering?. Phytother. Res. Ptr.

[73] Wang Z.C., Sun H.J., Li K.H., Fu C., Liu M.Z. (2014). Icariin promotes directed chondrogenic differentiation of bone marrow mesenchymal stem cells but not hypertrophy in vitro. Exp. Med..

[74] Zhang L., Zhang X., Li K.F., Li D.X., Xiao Y.M., Fan Y.J., Zhang X.D. (2012). Icariin promotes extracellular matrix synthesis and gene expression of chondrocytes in vitro. Phytother. Res. Ptr.

[75] Liu M.H., Sun J.S., Tsai S.W., Sheu S.Y., Chen M.H. (2010). Icariin protects murine chondrocytes from lipopolysaccharide-induced inflammatory responses and extracellular matrix degradation. Nutr. Res. (N.Y.).

[76] Xu C.Q., Liu B.J., Wu J.F., Xu Y.C., Duan X.H., Cao Y.X., Dong J.C. (2010). Icariin attenuates LPS-induced acute inflammatory responses: Involvement of PI3K/Akt and NF-kappaB signaling pathway. Eur. J. Pharm..

[77] Liu N., Zhang T., Cao B.R., Luan F.Y., Liu R.X., Yin H.R., Wang W.B. (2018). Icariin possesses chondroprotective efficacy in a rat model of dexamethasone-induced cartilage injury through the activation of miR-206 targeting of cathepsin K. Int. J. Med. Mol. Adv. Sci..

[78] Wang W., Sun L., Zhang P., Song J., Liu W. (2014). An anti-inflammatory cell-free collagen/resveratrol scaffold for repairing osteochondral defects in rabbits. Acta Biomater..

[79] Kankala R.K., Lu F.J., Liu C.G., Zhang S.S., Chen A.Z., Wang S.B. (2018). Effect of Icariin on Engineered 3D-Printed Porous Scaffolds for Cartilage Repair. Materials..

[80] Yang J., Liu Y., He L., Wang Q., Wang L., Yuan T., Xiao Y., Fan Y., Zhang X. (2018). Icariin conjugated hyaluronic acid/collagen hydrogel for osteochondral interface restoration. Acta Biomater..

[81] Li D., Yuan T., Zhang X., Xiao Y., Wang R., Fan Y., Zhang X. (2012). Icariin: A potential promoting compound for cartilage tissue engineering. Osteoarthr. Cartil..

[82] Wang P., Zhang F., He Q., Wang J., Shiu H.T., Shu Y., Tsang W.P., Liang S., Zhao K., Wan C. (2016). Flavonoid compound icariin activates Hypoxia Inducible Factor-1α in chondrocytes and promotes articular cartilage repair. PLoS ONE.

[83] Luo Y., Zhang Y., Huang Y. (2018). Icariin reduces cartilage degeneration in a mouse model of osteoarthritis and is associated with the changes in expression of Indian Hedgehog and Parathyroid Hormone-Related protein. Med Sci. Monit. Int. Med. J. Exp. Clin. Res..

[84] Liu Y., Yang J., Luo Z., Li D., Lu J., Wang Q., Xiao Y., Zhang X. (2019). Development of an injectable thiolated icariin functionalized collagen/hyaluronic hydrogel to promote cartilage formation in vitro and in vivo. J. Mater. Chem. B.

[85] Zhang J., Zhang D., Wu C., Liu A., Zhang C., Jiao J., Shang M. (2019). Icariin-conditioned serum engineered with hyaluronic acid promote repair of articular cartilage defects in rabbit knees. BMC Complementary Altern. Med..

[86] Cameron M., Chrubasik S. (2014). Oral herbal therapies for treating osteoarthritis. Cochrane Database Syst. Rev..

[87] Salehi B., Rescigno A., Dettori T., Calina D., Docea A.O., Singh L., Cebeci F., Ozcelik B., Bhia M., Dowlati Beirami A. (2020). Avocado-Soybean unsaponifiables: A panoply of potentialities to be exploited. Biomolecules.

[88] Henrotin Y.E., Labasse A.H., Jaspar J.M., De Groote D.D., Zheng S.X., Guillou G.B., Reginster J.Y. (1998). Effects of three avocado/soybean unsaponifiable mixtures on metalloproteinases, cytokines and prostaglandin E2 production by human articular chondrocytes. Clin. Rheumatol..

[89] Henrotin Y.E., Sanchez C., Deberg M.A., Piccardi N., Guillou G.B., Msika P., Reginster J.Y. (2003). Avocado/soybean unsaponifiables increase aggrecan synthesis and reduce catabolic and proinflammatory mediator production by human osteoarthritic chondrocytes. J. Rheumatol..

[90] Khanna D., Sethi G., Ahn K.S., Pandey M.K., Kunnumakkara A.B., Sung B., Aggarwal A., Aggarwal B.B. (2007). Natural products as a gold mine for arthritis treatment. Curr. Opin. Pharm..

[91] Hashemibeni B., Valiani A., Esmaeli M., Kazemi M., Aliakbari M., Iranpour F.G. (2018). Comparison of the efficacy of piascledine and transforming growth factor beta1 on chondrogenic differentiation of human adipose-derived stem cells in fibrin and fibrin-alginate scaffolds. Iran. J. Basic Med. Sci..

[92] Altinel L., Saritas Z.K., Kose K.C., Pamuk K., Aksoy Y., Serteser M. (2007). Treatment with unsaponifiable extracts of avocado and soybean increases TGF-beta1 and TGF-beta2 levels in canine joint fluid. Tohoku J. Exp. Med..

[93] Boumediene K., Felisaz N., Bogdanowicz P., Galera P., Guillou G.B., Pujol J.P. (1999). Avocado/soya unsaponifiables enhance the expression of transforming growth factor beta1 and beta2 in cultured articular chondrocytes. Arthritis Rheum..

[94] Maheu E., Mazières B., Valat J.P., Loyau G., Le Loët X., Bourgeois P., Grouin J.M., Rozenberg S. (1998). Symptomatic efficacy of avocado/soybean unsaponifiables in the treatment of osteoarthritis of the knee and hip: A prospective, randomized, double-blind, placebo-controlled, multicenter clinical trial with a six-month treatment period and a two-month followup demonstrating a persistent effect. Arthritis Rheum..

[95] Frondoza C.G., Fortuno L.V., Grzanna M.W., Ownby S.L., Au A.Y., Rashmir-Raven A.M. (2018). α-Lipoic acid potentiates the anti-inflammatory activity of avocado/soybean unsaponifiables in chondrocyte cultures. Cartilage.

[96] Au R.Y., Al-Talib T.K., Au A.Y., Phan P.V., Frondoza C.G. (2007). Avocado soybean unsaponifiables (ASU) suppress TNF-alpha, IL-1beta, COX-2, iNOS gene expression, and prostaglandin E2 and nitric oxide production in articular chondrocytes and monocyte/macrophages. Osteoarthr. Cartil..

[97] Lippiello L., Nardo J.V., Harlan R., Chiou T. (2008). Metabolic effects of avocado/soy unsaponifiables on articular chondrocytes. Evid.-Based Complementary Altern. Med.: Ecam.

[98] Cake M.A., Read R.A., Guillou B., Ghosh P. (2000). Modification of articular cartilage and subchondral bone pathology in an ovine meniscectomy model of osteoarthritis by avocado and soya unsaponifiables (ASU). Osteoarthr. Cartil..

[99] Boileau C., Martel-Pelletier J., Caron J., Msika P., Guillou G.B., Baudouin C., Pelletier J.P. (2009). Protective effects of total fraction of avocado/soybean unsaponifiables on the structural changes in experimental dog osteoarthritis: Inhibition of nitric oxide synthase and matrix metalloproteinase-13. Arthritis Res. Ther..

[100] Fazelipour S., Jahromy M.H., Tootian Z., Kiaei S.B., Sheibani M.T., Talaee N. (2012). The effect of chronic administration of methylphenidate on morphometric parameters of testes and fertility in male mice. J. Reprod. Infertil..

[101] Afaq F., Malik A., Syed D., Maes D., Matsui M.S., Mukhtar H. (2005). Pomegranate fruit extract modulates UV-B-mediated phosphorylation of mitogen-activated protein kinases and activation of nuclear factor kappa B in normal human epidermal keratinocytes paragraph sign. Photochem. Photobiol..

[102] Gil M.I., Tomás-Barberán F.A., Hess-Pierce B., Holcroft D.M., Kader A.A. (2000). Antioxidant activity of pomegranate juice and its relationship with phenolic composition and processing. J. Agric. Food. Chem..

[103] Seeram N.P., Nair M.G. (2002). Inhibition of lipid peroxidation and structure-activity-related studies of the dietary constituents anthocyanins, anthocyanidins, and catechins. J. Agric. Food. Chem..

[104] Sreekumar S., Sithul H., Muraleedharan P., Azeez J.M., Sreeharshan S. (2014). Pomegranate fruit as a rich source of biologically active compounds. Biomed. Res. Int..

[105] Sharma P., McClees S.F., Afaq F. (2017). Pomegranate for prevention and treatment of cancer: An update. Molecules.

[106] Ajaikumar K.B., Asheef M., Babu B.H., Padikkala J. (2005). The inhibition of gastric mucosal injury by Punicagranatum, L. (pomegranate) methanolic extract. J. Ethnopharmacol..

[107] Akhtar N., Haqqi T.M. (2012). Current nutraceuticals in the management of osteoarthritis: A review. Ther. Adv. Musculoskelet. Dis..

[108] Seeram N.P., Adams L.S., Henning S.M., Niu Y., Zhang Y., Nair M.G., Heber D. (2005). In vitro antiproliferative, apoptotic and antioxidant activities of punicalagin, ellagic acid and a total pomegranate tannin extract are enhanced in combination with other polyphenols as found in pomegranate juice. J. Nutr. Biochem..

[109] Ahmed S., Wang N., Hafeez B.B., Cheruvu V.K., Haqqi T.M. (2005). Punica granatum L. extract inhibits IL-1beta-induced expression of matrix metalloproteinases by inhibiting the activation of MAP kinases and NF-kappaB in human chondrocytes in vitro. J. Nutr..

[110] Shukla M., Gupta K., Rasheed Z., Khan K.A., Haqqi T.M. (2008). Bioavailable constituents/metabolites of pomegranate (Punica granatum L) preferentially inhibit COX2 activity ex vivo and IL-1beta-induced PGE2 production in human chondrocytes in vitro. J. Inflamm. (Lond. Engl.).

[111] Haseeb A., Khan N.M., Ashruf O.S., Haqqi T.M. (2017). A polyphenol-rich pomegranate fruit extract euppresses NF-κB and IL-6 expression by blocking the activation of IKKβ and NIK in primary human chondrocytes. Phytother. Res. Ptr.

[112] Rasheed Z., Akhtar N., Haqqi T.M. (2010). Pomegranate extract inhibits the interleukin-1beta-induced activation of MKK-3, p38alpha-MAPK and transcription factor RUNX-2 in human osteoarthritis chondrocytes. Arthritis Res. Ther..

[113] Akhtar N., Khan N.M., Ashruf O.S., Haqqi T.M. (2017). Inhibition of cartilage degradation and suppression of PGE (2) and MMPs expression by pomegranate fruit extract in a model of posttraumatic osteoarthritis. Nutrition (Burbank Los Angeles Cty. Calif.).

[114] Hadipour-Jahromy M., Mozaffari-Kermani R. (2010). Chondroprotective effects of pomegranate juice on monoiodoacetate-induced osteoarthritis of the knee joint of mice. Phytother. Res. Ptr.

[115] Monsefi M., Parvin F., Talaei-Khozani T. (2012). Effects of pomegranate extracts on cartilage, bone and mesenchymal cells of mouse fetuses. Br. J. Nutr..

[116] Ghoochani N., Karandish M., Mowla K., Haghighizadeh M.H., Jalali M.T. (2016). The effect of pomegranate juice on clinical signs, matrix metalloproteinases and antioxidant status in patients with knee osteoarthritis. J. Sci. Food Agric..

[117] Shakibaei M., Harikumar K.B., Aggarwal B.B. (2009). Resveratrol addiction: To die or not to die. Mol. Nutr. Food Res..

[118] Takaoka M. (1939). Resveratrol, a new phenolic compound, from Veratrum grandiflorum. Nippon Kagaku Kaishi.

[119] Borriello A., Bencivenga D., Caldarelli I., Tramontano A., Borgia A., Zappia V., Della Ragione F. (2014). Resveratrol: From basic studies to bedside. Cancer Treat. Res..

[120] Csiszar A. (2011). Anti-inflammatory effects of resveratrol: Possible role in prevention of age-related cardiovascular disease. Ann. N. Y. Acad. Sci..

[121] Csaki C., Keshishzadeh N., Fischer K., Shakibaei M. (2008). Regulation of inflammation signalling by resveratrol in human chondrocytes in vitro. Biochem. Pharmacol..

[122] Shakibaei M., Csaki C., Nebrich S., Mobasheri A. (2008). Resveratrol suppresses interleukin-1beta-induced inflammatory signaling and apoptosis in human articular chondrocytes: Potential for use as a novel nutraceutical for the treatment of osteoarthritis. Biochem. Pharmacol..

[123] Dave M., Attur M., Palmer G., Al-Mussawir H.E., Kennish L., Patel J., Abramson S.B. (2008). The antioxidant resveratrol protects against chondrocyte apoptosis via effects on mitochondrial polarization and ATP production. Arthritis Rheum..

[124] Kim H.J., Braun H.J., Dragoo J.L. (2014). The effect of resveratrol on normal and osteoarthritic chondrocyte metabolism. Bone Jt. Res..

[125] Liu F.C., Hung L.F., Wu W.L., Chang D.M., Huang C.Y., Lai J.H., Ho L.J. (2010). Chondroprotective effects and mechanisms of resveratrol in advanced glycation end products-stimulated chondrocytes. Arthritis Res. Ther..

[126] Meng X., Zhou J., Zhao C.N., Gan R.Y., Li H.B. (2020). Health benefits and molecular mechanisms of resveratrol: A narrative review. Foods (Basel Switz.).

[127] Frémont L. (2000). Biological effects of resveratrol. Life Sci..

[128] Pezzuto J.M. (2019). Resveratrol: Twenty years of growth, development and controversy. Biomol. Ther..

[129] Mobasheri A., Shakibaei M. (2013). Osteogenic effects of resveratrol in vitro: Potential for the prevention and treatment of osteoporosis. Ann. N. Y. Acad. Sci..

[130] Xie W.L., Chipman J.G., Robertson D.L., Erikson R.L., Simmons D.L. (1991). Expression of a mitogen-responsive gene encoding prostaglandin synthase is regulated by mRNA splicing. Proc. Natl. Acad. Sci. USA.

[131] Tseng C.C., Chen Y.J., Chang W.A., Tsai W.C., Ou T.T., Wu C.C., Sung W.Y., Yen J.H., Kuo P.L. (2020). Dual Role of chondrocytes in rheumatoid arthritis: The chicken and the egg. Int. J. Mol. Sci..

[132] Varela-Eirin M., Loureiro J., Fonseca E., Corrochano S., Caeiro J.R., Collado M., Mayan M.D. (2018). Cartilage regeneration and ageing: Targeting cellular plasticity in osteoarthritis. Ageing Res. Rev..

[133] Subbaramaiah K., Chung W.J., Michaluart P., Telang N., Tanabe T., Inoue H., Jang M., Pezzuto J.M., Dannenberg A.J. (1998). Resveratrol inhibits cyclooxygenase-2 transcription and activity in phorbol ester-treated human mammary epithelial cells. J. Biol. Chem..

[134] Im H.J., Li X., Chen D., Yan D., Kim J., Ellman M.B., Stein G.S., Cole B., Kc R., Cs-Szabo G. (2012). Biological effects of the plant-derived polyphenol resveratrol in human articular cartilage and chondrosarcoma cells. J. Cell. Physiol..

[135] Eo S.H., Cho H., Kim S.J. (2013). Resveratrol inhibits nitric oxide-induced apoptosis via the NF-Kappa B pathway in rabbit articular chondrocytes. Biomol. Ther..

[136] Maepa M., Razwinani M., Motaung S. (2016). Effects of resveratrol on collagen type II protein in the superficial and middle zone chondrocytes of porcine articular cartilage. J. Ethnopharmacol..

[137] Elmali N., Esenkaya I., Harma A., Ertem K., Turkoz Y., Mizrak B. (2005). Effect of resveratrol in experimental osteoarthritis in rabbits. Inflamm. Res. Off. J. Eur. Histamine Res. Soc..

[138] Li W., Cai L., Zhang Y., Cui L., Shen G. (2015). Intra-articular resveratrol injection prevents osteoarthritis progression in a mouse model by activating SIRT1 and thereby silencing HIF-2α. J. Orthop. Res. Off. Publ. Orthop. Res. Soc..

[139] Buhrmann C., Busch F., Shayan P., Shakibaei M. (2014). Sirtuin-1 (SIRT1) is required for promoting chondrogenic differentiation of mesenchymal stem cells. J. Biol. Chem..

[140] Qin N., Wei L., Li W., Yang W., Cai L., Qian Z., Wu S. (2017). Local intra-articular injection of resveratrol delays cartilage degeneration in C57BL/6 mice by inducing autophagy via AMPK/mTOR pathway. J. Pharmacol. Sci..

[141] Jin H., Zhang H., Ma T., Lan H., Feng S., Zhu H., Ji Y. (2018). Resveratrol protects murine chondrogenic ATDC5 cells ggainst LPS-Induced inflammatory injury through up-regulating MiR-146b. Cell. Physiol. Biochem. Int. J. Exp. Cell. Physiol. Biochem. Pharmacol..

[142] Wang J., Gao J.S., Chen J.W., Li F., Tian J. (2012). Effect of resveratrol on cartilage protection and apoptosis inhibition in experimental osteoarthritis of rabbit. Rheumatol. Int..

[143] Lei M., Liu S.Q., Liu Y.L. (2008). Resveratrol protects bone marrow mesenchymal stem cell derived chondrocytes cultured on chitosan-gelatin scaffolds from the inhibitory effect of interleukin-1beta. Acta Pharmacol. Sin..

[144] Sheu S.Y., Chen W.S., Sun J.S., Lin F.H., Wu T. (2013). Biological characterization of oxidized hyaluronic acid/resveratrol hydrogel for cartilage tissue engineering. J. Biomed. Mater. Res. Part. A.

[145] Wu G., Wang L., Li H., Ke Y., Yao Y. (2016). Function of sustained released resveratrol on IL-1β-induced hBMSC MMP13 secretion inhibition and chondrogenic differentiation promotion. J. Biomater. Appl..

[146] Shakibaei M., Mobasheri A., Buhrmann C. (2011). Curcumin synergizes with resveratrol to stimulate the MAPK signaling pathway in human articular chondrocytes in vitro. Genes Nutr..

[147] EFSA, European Food Safety Authority www.efsa.europa.eu.

[148] FDA, US Food and Drug Administration www.fda.gov.

[149] AHP, American Herbal Pharmacopoeia Monographs https://herbal-ahp.org.

[150] ESCOP, European Scientific Cooperative on Phytotherapy Monographs https://escop.com.

